# Noninvasive interrogation of CD8^+^ T cell effector function for monitoring early tumor responses to immunotherapy

**DOI:** 10.1172/JCI161065

**Published:** 2022-08-15

**Authors:** Haoyi Zhou, Yanpu Wang, Hongchuang Xu, Xiuling Shen, Ting Zhang, Xin Zhou, Yuwen Zeng, Kui Li, Li Zhang, Hua Zhu, Xing Yang, Nan Li, Zhi Yang, Zhaofei Liu

**Affiliations:** 1 Medical Isotopes Research Center and Department of Radiation Medicine, School of Basic Medical Sciences, Peking University Health Science Center, Beijing, China.; 2 Department of Nuclear Medicine, |Peking University First Hospital, Beijing, China.; 3 Key Laboratory of Carcinogenesis and Translational Research (Ministry of Education/Beijing), Department of Nuclear Medicine, and; 4 Department of Pathology, Peking University Cancer Hospital and Institute, Beijing, China.

**Keywords:** Oncology, Cancer immunotherapy, Diagnostic imaging

## Abstract

Accurately identifying patients who respond to immunotherapy remains clinically challenging. A noninvasive method that can longitudinally capture information about immune cell function and assist in the early assessment of tumor responses is highly desirable for precision immunotherapy. Here, we show that PET imaging using a granzyme B–targeted radiotracer named ^68^Ga-grazytracer, could noninvasively and effectively predict tumor responses to immune checkpoint inhibitors and adoptive T cell transfer therapy in multiple tumor models. ^68^Ga-grazytracer was designed and selected from several radiotracers based on non-aldehyde peptidomimetics, and exhibited excellent in vivo metabolic stability and favorable targeting efficiency to granzyme B secreted by effector CD8^+^ T cells during immune responses. ^68^Ga-grazytracer permitted more sensitive discrimination of responders and nonresponders than did ^18^F-fluorodeoxyglucose, distinguishing between tumor pseudoprogression and true progression upon immune checkpoint blockade therapy in mouse models with varying immunogenicity. In a preliminary clinical trial with 5 patients, no adverse events were observed after ^68^Ga-grazytracer injection, and clinical responses in cancer patients undergoing immunotherapy were favorably correlated with ^68^Ga-grazytracer PET results. These results highlight the potential of ^68^Ga-grazytracer PET to enhance the clinical effectiveness of granzyme B secretion–related immunotherapies by supporting early response assessment and precise patient stratification in a noninvasive and longitudinal manner.

## Graphical abstract

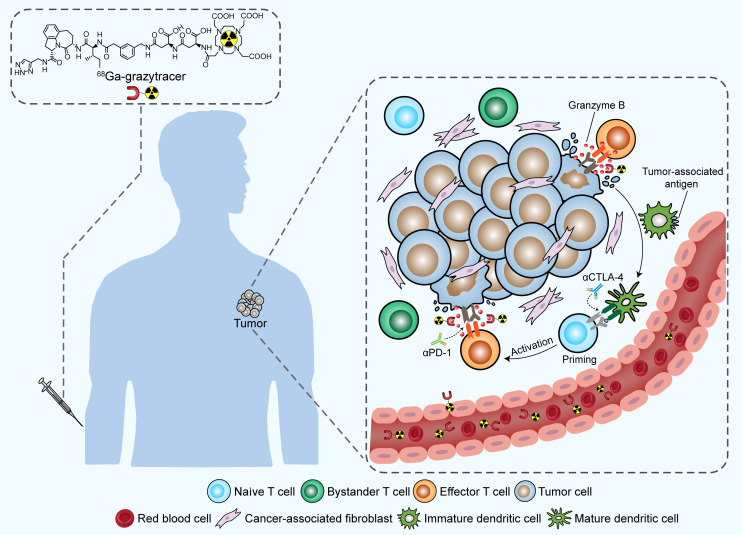

## Introduction

Immunotherapy — represented by immune checkpoint blockade using antibodies against cytotoxic T lymphocyte–associated protein 4 (CTLA-4), programmed cell death protein 1 (PD-1), and programmed cell death-ligand 1 (PD-L1) — has achieved remarkable clinical success in cancer therapy (1, 2). However, the relatively small fraction of patients who benefit from immune checkpoint inhibitors (ICIs; typically <30%), as well as immunotherapy-related adverse events (irAEs), limits its widespread application for the treatment of solid tumors (3, 4). Therefore, development of reliable approaches to monitor and evaluate tumor responses to immunotherapies at an early stage is critical to facilitate precise patient stratification.

Assessment of immunotherapy efficacy using conventional CT- and MRI-based response evaluation criteria in solid tumors (RECIST) has certain inherent limitations. In particular, it is difficult to distinguish whether increased tumor size is due to disease progression or pseudoprogression caused by immunotherapy-induced immune cell infiltration into the tumors (typically a positive indicator for tumor response to treatment) (5–7). Thus, CT- and MRI-based immune-related response criteria (irRC) and immunotherapy RECIST (iRECIST) standards have been developed to improve the discrimination between true disease progression and pseudoprogression for patients treated with immunotherapy (6, 8, 9). However, such anatomical imaging–based evaluation criteria cannot provide early response assessment and cannot confirm disease progression until at least 4 weeks after onset of immunotherapy, which may be beyond the period when treatment strategies can be effectively adjusted (10, 11).

As a noninvasive whole-body imaging modality that can capture biological processes at the molecular level, PET has been extensively used for clinical cancer diagnosis, staging, patient stratification, and monitoring tumor responses to therapies (12, 13). Uptake of ^18^F-fluorodeoxyglucose (^18^F-FDG), the PET radiotracer most commonly used in the clinic, reflects the glucose metabolism of cells within the tumor area. However, ^18^F-FDG cannot differentiate between proliferative tumor cells and immunotherapy-induced tumor-infiltrating immune cells, which significantly reduces its specificity for immunotherapy-related applications (14, 15). Immune-related biomarker-specific PET radiotracers have thus been investigated for noninvasive visualization of biomarkers before or during immunotherapy to predict and/or monitor tumor responses. For example, in clinical trials, radiolabeled antibodies targeting PD-1 and PD-L1 have been investigated to assess tumor responses to PD-1/PD-L1 therapy. These studies have shown that radiotracer uptake before therapy correlated well with treatment response and patient survival (16, 17). However, the predictive value of PD-L1 is typically limited to specific cases and must therefore be implemented with caution (18). Radiotracers targeting CD8 have also been investigated for preclinical PET assessment of ICI therapy (19, 20). However, CD8-targeted PET cannot distinguish resident from infiltrating CD8^+^ T cells, or activated from nonactivated populations (21). Alternative biomarkers, such as T cell activation biomarkers, including OX40 and ICOS, have recently been studied for PET monitoring of tumor responses to immunotherapy (15, 22). Although these methods could indicate the early activation state of T cells, expression of these molecules on immunosuppressive cells such as Tregs is also high, which might interfere with the analysis of imaging results (23). Moreover, most of these radiotracers were developed in preclinical settings using antibodies as targeting vehicles; this necessitates several days in order to clear the background signal, which would make it difficult for same-day clinical use in PET.

T cell effector function in immunotherapy relies on the release of perforin and granzyme B, which enter tumor cells and trigger the caspase cascade, leading to tumor cell death. An attractive immune-related biomarker, granzyme B secretion represents the final signal of multiple antitumor immune pathways, not only reflecting the localization of cytotoxic T cells in the tumor area, but also directly indicating the potential ability of cytotoxic T cells to kill tumor cells (24). Therefore, PET of granzyme B may facilitate direct visualization of tumor killing by immune cells, suggesting that it is a reliable biomarker of tumor responses to immunotherapy. Pioneering studies have recently been conducted to develop a ^68^Ga-labeled linear peptide GZP–based granzyme B–targeting radiotracer, ^68^Ga-1,4,7–triazacyclononane-1,4,7-triacetic acid–GZP (^68^Ga-NOTA-GZP), for PET monitoring of tumor immunotherapy in mouse models (25–27). GZP was designed from a cleavage tetrapeptide sequence (Ile-Glu-Phe-Asp) for murine granzyme B (28, 29) with a C-terminal aldehyde modification to induce irreversible binding (25). As it is a linear peptide aldehyde, the tumor uptake of ^68^Ga-NOTA-GZP was low, most likely due to the inherent instability of linear peptides in vivo, and GZP may cross-react with other serine proteases on the reactive aldehyde pharmacophore (30). In addition, as the Ile-Glu-Phe-Asp sequence is more specific to murine than human granzyme B (29, 31), the results with ^68^Ga-NOTA-GZP obtained from mouse models may not be directly translatable to clinical situations.

To develop a clinically translatable radiotracer for annotating T cell effector function, in this study we designed and synthesized several granzyme B–targeting precursors based on non-aldehyde peptidomimetics (30). We hypothesized that the rigid tricyclic peptidomimetic scaffold might help improve in vivo stability, while the non-aldehyde pharmacophore could eliminate the possibility of reacting with other proteases. One lead compound was identified and radiolabeled with ^68^Ga to generate the radiotracer, designated as ^68^Ga-grazytracer. ^68^Ga-grazytracer showed binding patterns similar to those of murine and human granzyme B proteins, suggesting that this radiotracer may be readily translated from animal research to clinical practice. The role of ^68^Ga-grazytracer in monitoring early tumor responses to ICIs and adoptive T cell transfer (ACT) therapy, as well as differentiation of tumor pseudoprogression upon ICI treatment, was then investigated in preclinical mouse models. Moreover, a preliminary clinical trial of this radiotracer was performed in patients with lung cancer and melanoma to explore the potential application of ^68^Ga-grazytracer for the early identification of patient outcomes from immunotherapy.

## Results

### Limitations of ^18^F-FDG PET in detecting immunotherapy-induced tumor pseudoprogression.

**Figure 1:**
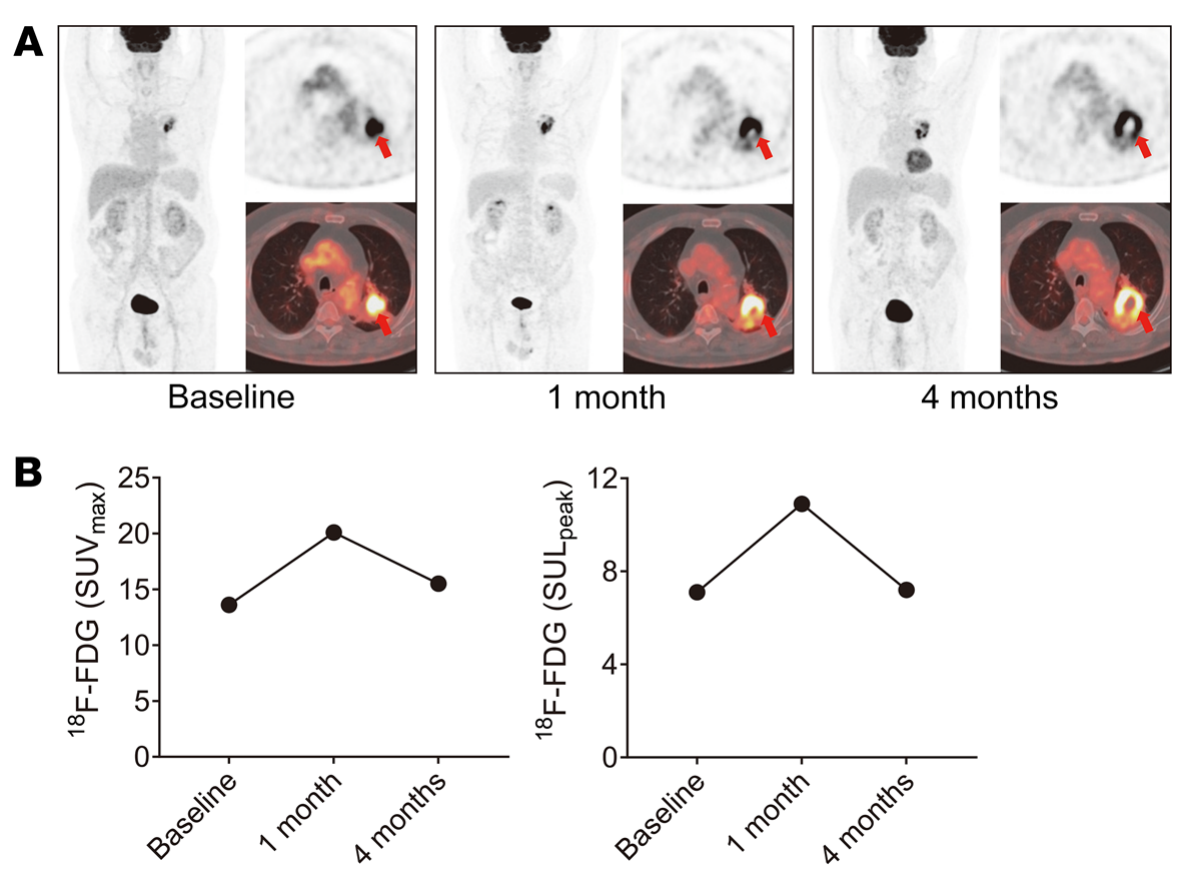
^18^F-FDG PET/CT images of a representative case of tumor pseudoprogression after ICI therapy. (**A**) The representative case was a 61-year-old man with lung squamous cell carcinoma (clinical stage cT3N1M0) receiving ipilimumab plus nivolumab. Baseline ^18^F-FDG PET/CT shows the SUV_max_ of the mass to be 13.6 and the SUL_peak_ to be 7.1. Interim PET/CT after 1 cycle of immunotherapy (1 month) shows that ^18^F-FDG uptake of the mass was increased, with a SUV_max_ of 20.1 and SUL_peak_ of 10.9 (PMD with PERCIST criteria). PET/CT after 3 cycles of therapy (4 months) shows that the ^18^F-FDG uptake in the mass decreased to a SUV_max_ of 15.5 and SUL_peak_ of 7.2 (PMR with PERCIST criteria). Tumors are indicated by red arrows. (**B**) The SUV_max_ and SUL_peak_ of ^18^F-FDG PET/CT at different stages of immunotherapy.

Immunotherapy drugs, such as ICIs, rely on immune cell infiltration in the tumor in order to function. ^18^F-FDG uptake reflects the glucose metabolism of cells; however, it does not effectively differentiate between enhanced glucose metabolism in proliferating tumor cells (true progressive disease) and that in infiltrating immune cells (pseudoprogression) (32). In an illustrative case in this study (Figure 1A), a 61-year-old man with a 5.3 × 3.6 cm mass located in the posterior apical segment of the upper lobe of the left lung was diagnosed with lung squamous cell carcinoma, clinical stage cT3N1M0. Baseline ^18^F-FDG PET/CT prior to immunotherapy with ipilimumab plus nivolumab revealed that the mass had a maximum standardized uptake value (SUV_max_) of 13.6 and peak SUV corrected for lean body mass (SUL_peak_) of 7.1. Compared with baseline, the SUV_max_ (value of 20.1) and SUL_peak_ (value of 10.9) of ^18^F-FDG in the early stage of 1 cycle of immunotherapy (1 month after immunotherapy) markedly increased. This was identified as a progressive metabolic disease (PMD) according to PET Response Criteria in Solid Tumors (PERCIST). However, following the completion of 3 immunotherapy cycles in 4 months, the SUV_max_ and SUL_peak_ of ^18^F-FDG PET/CT decreased to a level similar to that at baseline (15.5 and 7.2, respectively), which was identified as a partial metabolic response (PMR) using PERCIST (Figure 1B). This case represents a typical immunotherapy-related pseudoprogression in the interim evaluation; early ^18^F-FDG PET/CT (e.g., 1 month after immunotherapy) was limited in accurately defining true progression and pseudoprogression.

### Design and characterization of the granzyme B–targeting radiotracer ^68^Ga-grazytracer.

**Figure 2:**
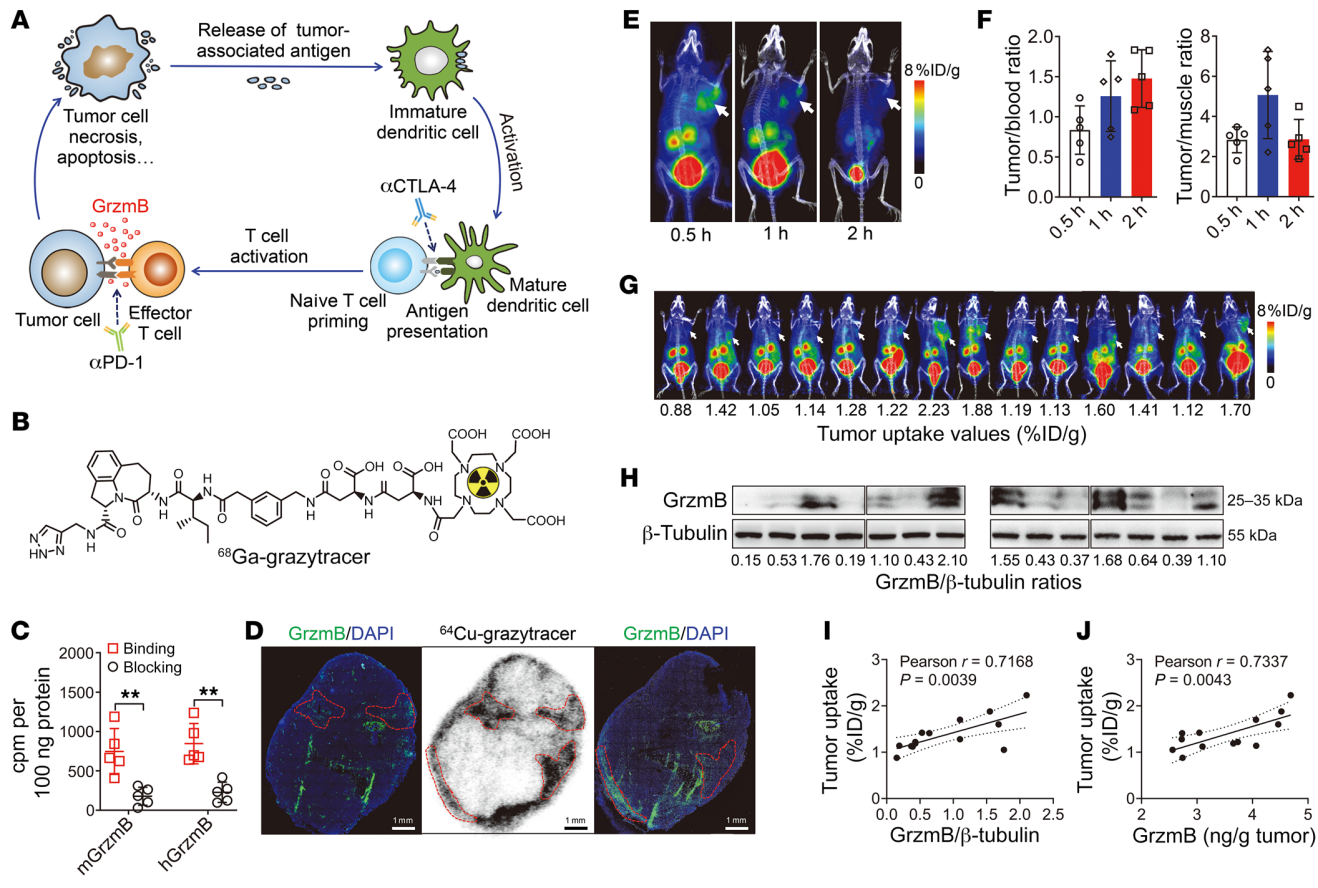
In vitro and in vivo characterization of ^68^Ga-grazytracer. (**A**) Schematic of effector T cell activation and granzyme B (GrzmB) secretion following immunotherapy. (**B**) Chemical structure of ^68^Ga-grazytracer. (**C**) Binding specificity of ^68^Ga-grazytracer with murine (m) or human (h) granzyme B (*n* = 5). (**D**) Autoradiography (middle) and granzyme B immunofluorescence staining (2 slides) of tumor serial sections (15 μm thick) harvested from MC38 tumor–bearing mice at 0.5 hours after ^64^Cu-grazytracer injection. The overlay regions in these 3 serial sections are indicated by dotted red lines. Scale bars: 1 mm. Data are representative of 3 independent experiments. (**E**) Representative PET images of ^68^Ga-grazytracer in anti–PD-1–treated mice at 0.5, 1, and 2 hours after injection. (**F**) Calculated tumor-to-blood and tumor-to-muscle ratios of ^68^Ga-grazytracer (*n* = 5). (**G**) Small-animal PET images of ^68^Ga-grazytracer and corresponding tumor uptake values in MC38 tumor–bearing mice (16 mice were subjected to PET imaging, and 2 were excluded due to failed tail vein injection). (**H**) Western blotting of granzyme B in MC38 tumors harvested from MC38 tumor–bearing mice (from **G**). The lanes were run on the same gel but were noncontiguous. (See supplemental material for full, uncut gels.) (**I**) Correlation between the tumor uptake of ^68^Ga-grazytracer quantified by PET imaging and granzyme B/β-tubulin ratios determined by ex vivo Western blotting (*r* = 0.7168 by Pearson’s correlation analysis). (**J**) Correlation between the tumor uptake of ^68^Ga-grazytracer quantified by PET imaging and ex vivo granzyme B levels determined by ELISA (*r* = 0.7337 by Pearson’s correlation analysis). Tumors are indicated by white arrows in PET images. All numerical data are presented as mean ± SD. **P < 0.01 by unpaired Student’s *t* test (**C**).

Given the insufficient capacity of ^18^F-FDG PET to assess tumor response during the early stages of immunotherapy, we sought to develop radiotracers targeting granzyme B that were secreted by effector CD8^+^ cytotoxic T lymphocytes (CTLs) during immunotherapy (Figure 2A). The 1,2,3-triazole–based non-aldehyde granzyme B inhibitor (Supplemental Figure 1A; supplemental material available online with this article; https://doi.org/10.1172/JCI161065DS1) was optimized from a granzyme B–targeting tetrapeptide aldehyde (Ile-Glu-Pro-Asp) (28) initially identified from combinatorial library screening. It showed great potency and selectivity for granzyme B, with a *K_i_* reaching 13 nM (30). Compared with Ile-Glu-Pro-Asp, the rigid tricyclic peptidomimetic scaffold of the 1,2,3-triazole–based non-aldehyde granzyme B inhibitor showed significantly improved potency and metabolic stability. Moreover, use of a 1,2,3-triazole moiety in place of an aldehyde makes it a more selective granzyme B inhibitor than other serine proteases, such as caspase-3 (28). Based on the peptidomimetic scaffold, we designed and synthesized 4 precursors for granzyme B targeting. These precursors contained substitutions away from the 1,2,3-triazole pharmacophore and included a 1,4,7,10-tetraazacyclododecane-1,4,7,10-tetraacetic acid (DOTA) for radiolabeling and various linkers to adjust hydrophilicity.

Next, MC38 tumor–bearing mice were pretreated with an anti–PD-1 antibody to induce the release of granzyme B in the tumors upon T cell activation. Small-animal PET imaging and biodistribution studies of the 4 radiotracers were performed. Among the 4, radiotracer 3 exhibited the best properties, including low liver and gallbladder uptake and high tumor/muscle ratio (Supplemental Figure 1, B–E). Ex vivo necropsy-based biodistribution experiments further confirmed the in vivo PET imaging results (Supplemental Figure 2, A–D). Therefore, radiotracer 3 (designated as ^68^Ga-grazytracer; Figure 2B) was selected for further studies. We found this radiotracer to be efficiently radiolabeled with ^68^Ga, with a decay-corrected radiochemical yield ranging from 95% to 98% and a radiochemical purity of 99% after purification. The specific activity and molar activity of purified ^68^Ga-grazytracer were calculated to be 13.1–13.6 MBq/μg and 15.8–16.3 MBq/nmol, respectively. ^68^Ga-grazytracer exhibited favorable in vitro stability, with a radiochemical purity of more than 99% after 2 hours (Supplemental Figure 3, A and B). Moreover, ^68^Ga-grazytracer showed favorable in vivo metabolic stability, with no metabolites in the blood or urine observed at 0.5 hours after injection (Supplemental Figure 4).

We further determined the binding specificity of ^68^Ga-grazytracer to granzyme B using an in vitro binding assay. The binding values of ^68^Ga-grazytracer to murine granzyme B and human granzyme B were significantly inhibited following addition of excess doses of unlabeled precursor 3 (described in Supplemental Methods) (*P* < 0.01; Figure 2C), suggesting receptor-mediated specific binding of the radiotracer. Autoradiography was also performed to investigate in vivo tumor localization of ^68^Ga-grazytracer after injection. Considering the short half-life of ^68^Ga (*t_1/2_* = 68 minutes), we radiolabeled precursor 3 with ^64^Cu (*t_1/2_* = 12.7 hours), which functioned as a surrogate for autoradiography studies. The distribution of ^64^Cu-grazytracer was consistent with the location of granzyme B, as determined using immunofluorescence staining of adjacent tumor sections (Figure 2D). Moreover, owing to the heterogeneous spatial distribution of granzyme B in the tumor, differential immunofluorescence staining of granzyme B in serial slices was observed (Figure 2D).

Granzyme B is a protein secreted by effector immune cells that can be released into the bloodstream, where it can affect the tumor targeting of ^68^Ga-grazytracer. Thus, we performed ELISA to estimate the fraction of granzyme B in the tumor versus circulation of tumor-bearing mice. The results showed that although granzyme B is a secretory protein, most of it was retained in the tumor site (93.81%) rather than distributed throughout the circulation (Supplemental Figure 5), suggesting the feasibility of in vivo imaging of granzyme B released by CTLs in the tumor.

Thus, we further investigated the in vivo distribution of ^68^Ga-grazytracer in MC38 tumor–bearing mice pretreated with anti–PD-1. ^68^Ga-grazytracer uptake was highest 0.5 hours after injection and decreased over time (Figure 2E). Tumor-to-blood ratios increased over time owing to the rapid clearance of the radiotracer from the blood. The highest tumor-to-muscle ratio was observed 1 hour after injection (Figure 2F). Ex vivo biodistribution confirmed the in vivo ^68^Ga-grazytracer PET results (Supplemental Figure 6).

To further investigate whether tumor uptake values of ^68^Ga-grazytracer PET can indeed reflect granzyme B levels in vivo, we performed PET imaging of ^68^Ga-grazytracer in 16 tumor-bearing mice. The mice were euthanized immediately after PET scanning, and granzyme B expression was measured in tumors (Figure 2, G and H). We observed a high positive correlation between ^68^Ga-grazytracer tumor uptake values determined via in vivo PET imaging and granzyme B levels quantified by ex vivo Western blotting (Pearson’s *r* = 0.7168, *P* < 0.01) and ELISA (Pearson’s *r* = 0.7337, *P* < 0.01) (Figure 2, I and J). These results suggested that ^68^Ga-grazytracer PET quantitatively detected granzyme B levels in vivo.

^68^Ga-NOTA-GZP (Supplemental Figure 7A) is a previously reported peptide-based radiotracer for granzyme B PET in vivo in animal models (25–27, 33). ^68^Ga-NOTA-GZP showed favorable stability in the blood at 0.5 hours after injection; however, we observed some decomposition in urine (Supplemental Figure 7B). For in vivo PET imaging, ^68^Ga-grazytracer had significantly higher tumor uptake and tumor contrast (tumor-to-muscle ratio) than ^68^Ga-NOTA-GZP (*P* < 0.05; Supplemental Figure 8, A–C) at 0.5 hours after injection, which might have been due to the superior metabolic stability of ^68^Ga-grazytracer compared with ^68^Ga-NOTA-GZP (Supplemental Figure 4 and Supplemental Figure 7B). Taken together, the results indicated that ^68^Ga-grazytracer exhibited favorable granzyme B–targeting efficiency and specificity, which could be utilized for further assessment in immunotherapy monitoring.

### PET of ^68^Ga-grazytracer enables early prediction of tumor responses to ICIs.

**Figure 3:**
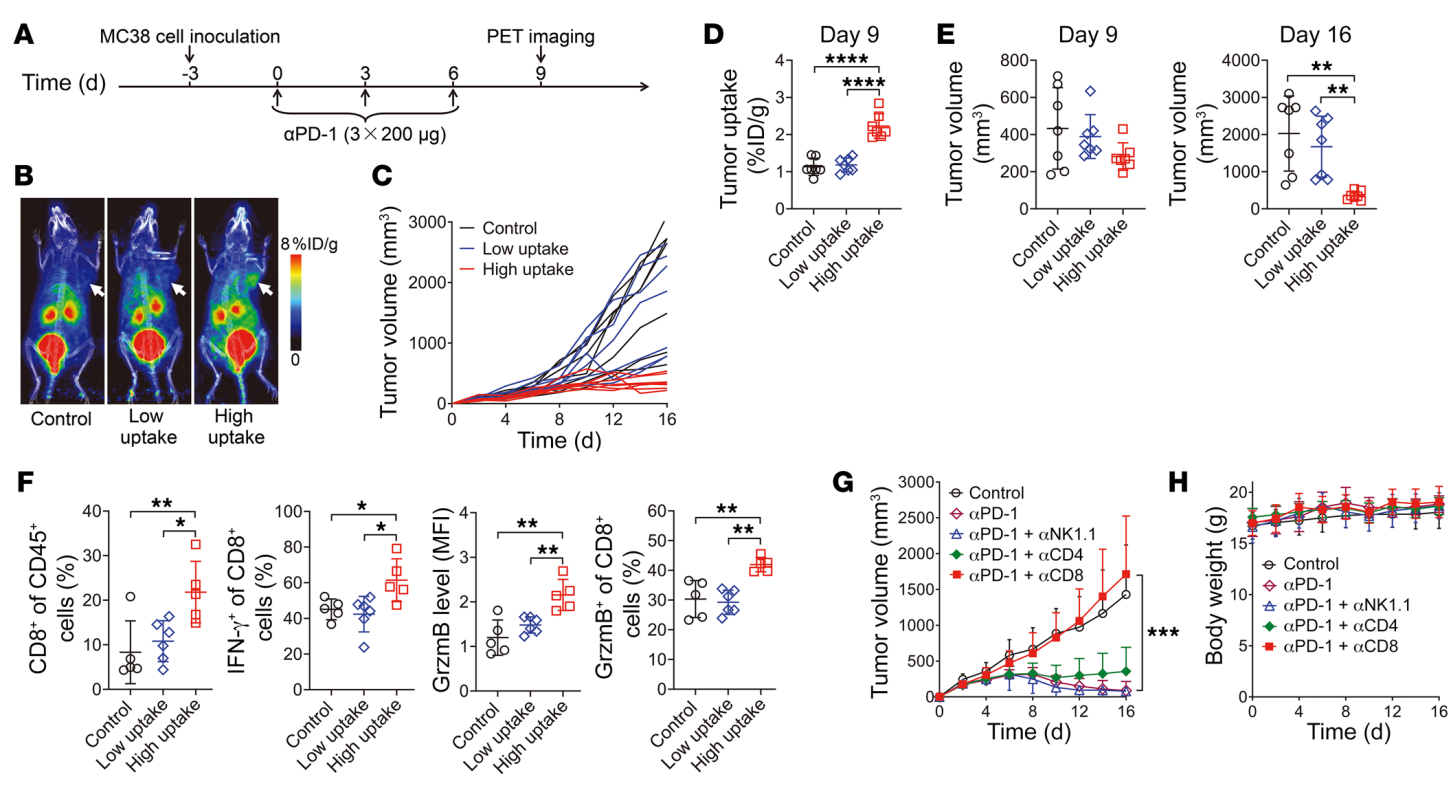
Small-animal PET imaging of ^68^Ga-grazytracer to predict tumor responses to anti–PD-1 therapy in MC38 tumor–bearing mice. (**A**) Timeline of anti–PD-1 (αPD-1) therapy and PET imaging in MC38 tumor–bearing mice. (**B**) Representative PET images of ^68^Ga-grazytracer at 0.5 hours after injection in MC38 tumor–bearing mice treated with PBS (control) or anti–PD-1 with high and low tumor uptake (cutoff of 1.45 %ID/g). Tumors are indicated by white arrows. (**C**) Individual tumor volumes of MC38 tumor–bearing mice in the control group and treatment groups with high and low tumor uptake. (**D**) Quantified tumor uptake of ^68^Ga-grazytracer at 0.5 hours after injection on day 9 in each group of MC38 tumor–bearing mice (*n =* 7/group). (**E**) Tumor volumes of MC38 tumor–bearing mice on days 9 and 16 (*n =* 7/group). (**F**) Flow cytometric analysis showing the proportion of CD8^+^ T cells in CD45^+^ cells, IFN-γ^+^ or granzyme B^+^ in CD8^+^ T cells, and the granzyme B levels in tumors harvested from mice after the indicated treatments (*n* = 5–6/group). (**G** and **H**) Tumor growth curves and body weight of MC38 tumor–bearing mice after the indicated treatments (*n =* 6–8/group). All numerical data are presented as mean ± SD. *P < 0.05, **P < 0.01, ***P < 0.001, ****P < 0.0001 by 1-way ANOVA with a post hoc Tukey’s test (**D**–**F**) and 2-way ANOVA (**G**).

Subsequently, we tested whether ^68^Ga-grazytracer PET could be used to predict early tumor responses to ICI in animal models by targeting granzyme B released by effector T cells upon immune responses. Fourteen MC38 tumor–bearing mice were treated with the anti–PD-1 antibody on days 0, 3, and 6, and ^68^Ga-grazytracer PET was conducted on day 9 (Figure 3A). Variable tumor uptake of ^68^Ga-grazytracer was observed in the treatment group (Figure 3B). The mice in the anti–PD-1–treated group were divided into 2 groups based on the tumor uptake values of ^68^Ga-grazytracer (cutoff of 1.45 percent injected dose per gram tissue [%ID/g] — the highest tumor uptake value of ^68^Ga-grazytracer in the control group): those with tumor uptake ≥1.45 %ID/g (high-uptake group; mean tumor uptake, 2.23 ± 0.33, range, 1.93–2.84); and those with tumor uptake <1.45 %ID/g (low-uptake group; mean tumor uptake, 1.18 ± 0.19, range, 0.93–1.44) (Figure 3B). Tumor growth curves for the vehicle control and anti–PD-1 treatment groups were monitored (Figure 3C). On day 9, the tumor uptake of ^68^Ga-grazytracer in the high-uptake group was significantly greater than that in the control and low-uptake groups (*P* < 0.0001; Figure 3D). However, at this time point, there were no significant differences in tumor volume among the control, low-uptake, and high-uptake groups (Figure 3E). On day 16, the tumor volume of the high-uptake group was considerably lower than that of the control and low-uptake groups (*P* < 0.01; Figure 3E). This result indicated that tumors with low ^68^Ga-grazytracer uptake did not respond to anti–PD-1 treatment, whereas tumors with high ^68^Ga-grazytracer uptake regressed to a significant degree (Figure 3, C and E). In a separate experiment, flow cytometric analysis of MC38 tumors on day 9 revealed an increased level of activated T cells (CD8^+^CD45^+^ T cells: 21.82% ± 6.91% vs. 10.81% ± 4.58% vs. 8.32% ± 7.05%; IFN-γ^+^CD8^+^ T cells: 61.42% ± 12.01% vs. 42.38% ± 9.95% vs. 45.08% ± 5.79%; granzyme B^+^CD8^+^ T cells: 41.94% ± 2.44% vs. 29.23% ± 4.06% vs. 30.32% ± 6.22%) and of granzyme B in the tumors of the high-uptake compared with the low-uptake and vehicle control groups (Figure 3F). These results demonstrated that ^68^Ga-grazytracer PET imaging could be effectively used to monitor the activation and granzyme B release of CD8^+^ CTLs to predict the potential efficacy of ICI. Similar results were obtained for ^68^Ga-grazytracer PET in mice bearing Lewis lung carcinoma (LLC) treated with anti–PD-1 and anti–CTLA-4 antibodies (Supplemental Figure 9, A–E).

Given the fact that NK cells, as an important component of innate immunity, can also kill tumor cells via granzyme B secretion (34), we performed an in vivo depletion study to verify the contribution of granzyme B secreted by NK cells and CD8^+^ CTLs during immunotherapy (Figure 3, G and H). Tumor growth inhibition of anti–PD-1 therapy was almost completely reversed in the CD8^+^ T cell depletion group (*P* < 0.001), while depletion of NK cells did not affect the therapeutic antitumor effect of anti–PD-1; this indicated that CD8^+^ T cells had a more critical role than NK cells in directing anti–PD-1 immunotherapy and that CD8^+^ T cells contributed more to the secretion of granzyme B in tumors treated with anti–PD-1 antibody.

### ^68^Ga-grazytracer PET allows for distinguishing pseudoprogression from true progression of tumors in animal models.

**Figure 4:**
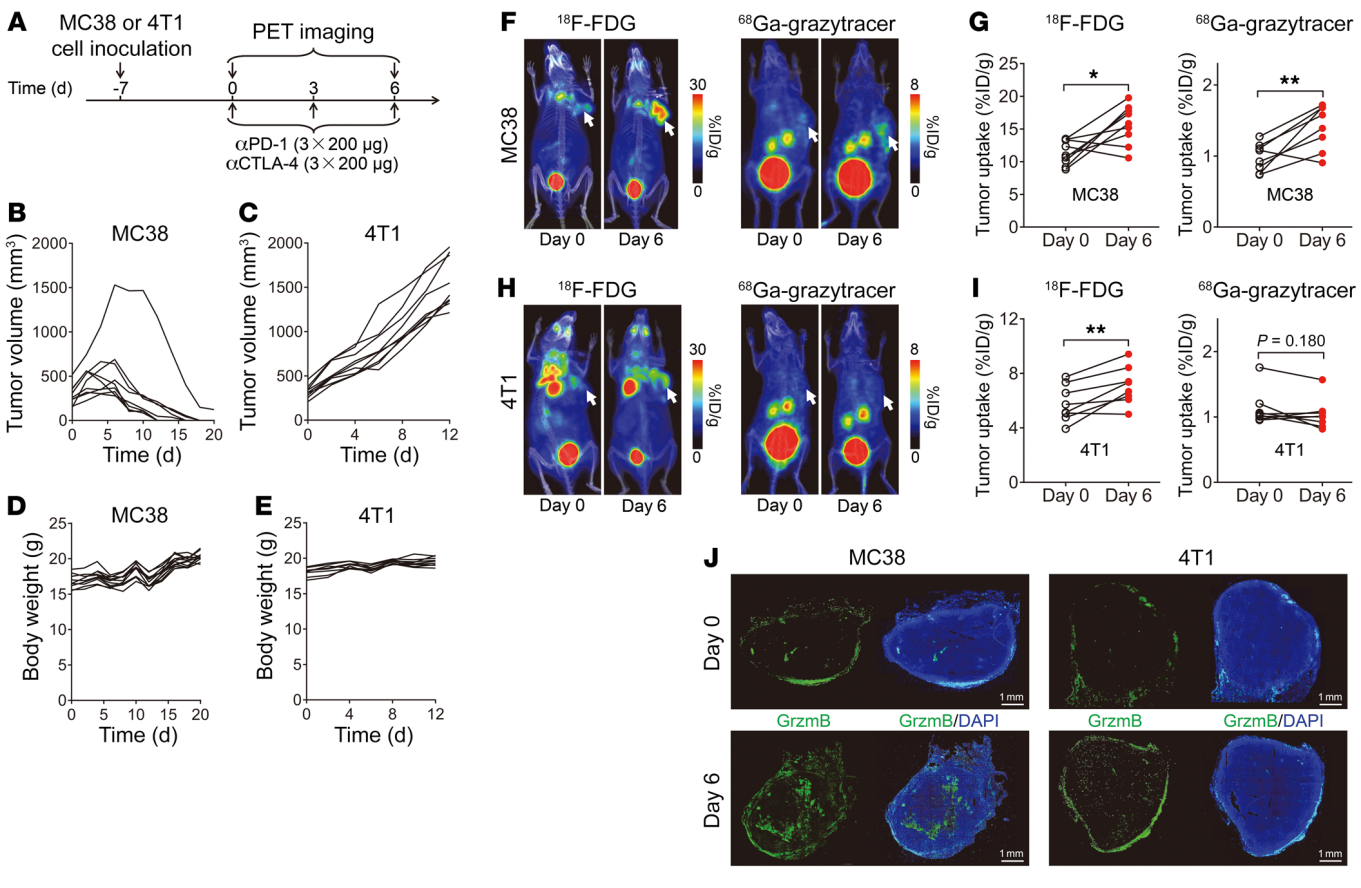
^68^Ga-grazytracer PET imaging in the pseudoprogression and true-progression murine models following treatment with anti–PD-1 and anti–CTLA-4. (**A**) Timeline of immunotherapy and PET imaging in MC38 or 4T1 tumor models. (**B** and **C**) Individual tumor growth curves of MC38 tumor–bearing mice (pseudoprogression) (**B**) and 4T1 tumor–bearing mice (true progression) (**C**) after treatment. (**D** and **E**) Body weight of MC38 (**D**) and 4T1 (**E**) tumor-bearing mice after treatment. (**F** and **G**) Representative PET images (**F**) and quantified tumor uptake (**G**) of ^18^F-FDG and ^68^Ga-grazytracer in pseudoprogression MC38 tumor–bearing mice on days 0 and 6 (*n =* 8–9/group). (**H** and **I**) Representative PET images (**H**) and quantified tumor uptake (**I**) of ^18^F-FDG and ^68^Ga-grazytracer in true-progression 4T1 tumor–bearing mice on days 0 and 6 (*n =* 8–9/group). (**J**) Representative immunofluorescence staining of granzyme B in MC38 or 4T1 tumor tissues harvested on days 0 and 6. Scale bars: 1 mm. Data are representative of 3 independent experiments. Tumors are indicated by white arrows in PET images. All numerical data are presented as mean ± SD. *P < 0.05, **P < 0.01 by 2-tailed paired Student’s *t* test (**G** and **I**).

To overcome the limitations of ^18^F-FDG (Figure 1), we explored the potential of ^68^Ga-grazytracer PET to distinguish tumor pseudoprogression from true progression via noninvasive imaging of granzyme B released by functional CTLs during ICI therapy. Hence we established mouse models bearing tumors with different immunogenicites — MC38 (highly immunogenic; ref. 35) and 4T1 (poorly immunogenic; ref. 36) — and treated the mice with anti–PD-1 and anti–CTLA-4 antibodies (Figure 4A). The tumor volume of the MC38 tumor–bearing mice increased during the initial stage of immunotherapy and reached a peak on approximately day 6 after treatment. Subsequently, the tumor volume rapidly decreased until tumor regression occurred (Figure 4B); thus, we considered this model to represent tumor pseudoprogression. In contrast, the 4T1 tumor–bearing mice showed complete progression upon anti–PD-1 plus anti–CTLA-4 treatment (Figure 4C), which we considered to represent a model of true tumor progression. In both models, the body weight of mice was not influenced by ICI treatment (Figure 4, D and E), suggesting that the toxicity elicited by this treatment strategy was limited.

We then performed PET imaging of ^18^F-FDG and ^68^Ga-grazytracer in the 2 tumor models on days 0 and 6. The tumor uptake of ^18^F-FDG in both the MC38 (*P* < 0.05) and 4T1 tumor (*P* < 0.01) models significantly increased from days 0 to 6 (Figure 4, F–I) owing to tumor progression (Figure 4, B and C) and increased glucose metabolism. However, while the tumor uptake of ^68^Ga-grazytracer significantly increased from days 0 to 6 in the pseudoprogression MC38 tumor mice (*P* < 0.01; Figure 4, F and G), no significant uptake of the ^68^Ga-grazytracer was observed in the true-progression 4T1 tumor group (Figure 4, H and I). These results suggest that ^68^Ga-grazytracer PET detected the high granzyme B secretion of the MC38 tumors but not that of the 4T1 tumors upon ICI therapy on day 6, thereby predicting the tumor responses at an early therapeutic stage. Ex vivo immunofluorescence staining confirmed the comparably low granzyme B secretion on day 0 in the 2 tumor models; however, considerably higher granzyme B secretion was observed in the MC38 compared with the 4T1 tumors on day 6 (Figure 4J), which was consistent with the ^68^Ga-grazytracer PET imaging results. Notably, immunofluorescence staining of NK1.1 showed that there was no significant change in infiltration of NK cells before and after treatment with anti–PD-1 plus anti–CTLA-4 in MC38 tumors (Supplemental Figure 10, A and B), which further confirmed that the changes in tumor uptake of ^68^Ga-grazytracer after ICI therapy was related to the secretion of granzyme B by CD8^+^ T cells. Collectively, these results indicated that ^68^Ga-grazytracer PET of CD8^+^ T cell effector function distinguished pseudoprogression from the true progression of ICI-treated tumors.

### ICI-induced tumor pseudoprogression is related to immune cell infiltration.

**Figure 5:**
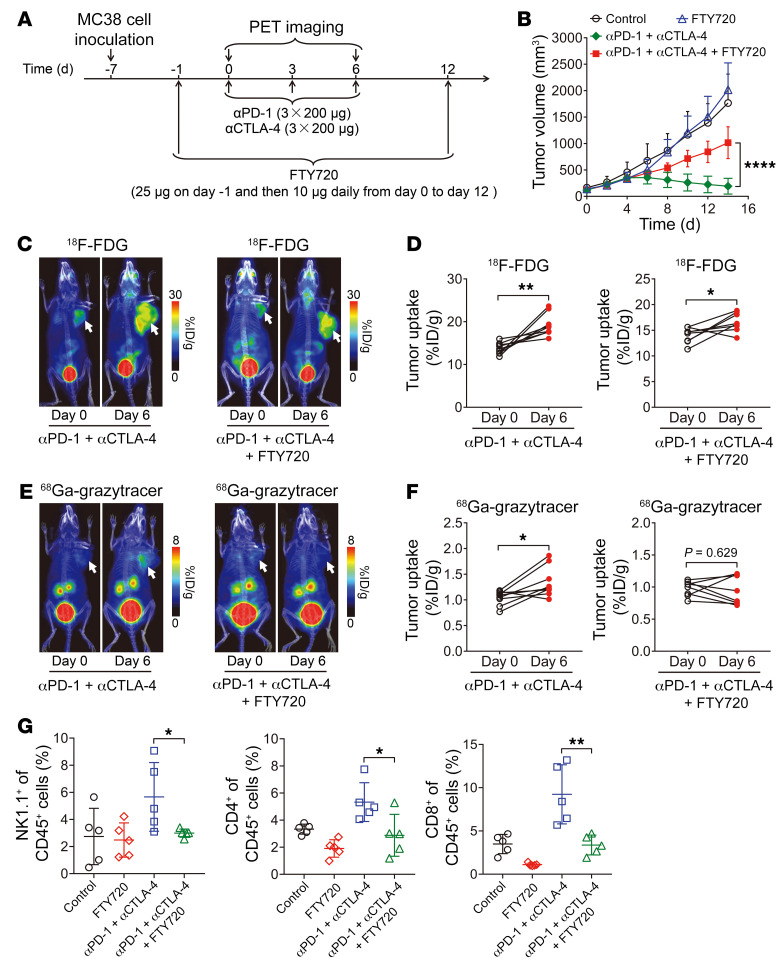
PET imaging of ^68^Ga-grazytracer in mouse models with or without inhibition of immune cell infiltration. (**A**) Timeline of PET imaging, anti–PD-1 plus anti–CTLA-4 combinational immunotherapy, and FTY720 treatment in MC38 tumor–bearing mice. (**B**) Tumor growth curves of MC38 tumor–bearing mice after the indicated treatments: control (PBS), FTY720, anti–PD-1 plus anti–CTLA-4, and anti–PD-1 plus anti–CTLA-4 plus FTY720 (*n =* 6–9/group). (**C** and **D**) Representative PET images (**C**) and quantified tumor uptake (**D**) of ^18^F-FDG on days 0 and 6 in anti–PD-1– plus anti–CTLA-4–treated MC38 tumor–bearing mice with or without FTY720 treatment (*n =* 8–9/group). (**E** and **F**) Representative PET images (**E**) and quantified tumor uptake (**F**) of ^68^Ga-grazytracer on days 0 and 6 in anti–PD-1– plus anti–CTLA-4–treated MC38 tumor–bearing mice with or without FTY720 treatment (*n =* 8–9/group). (**G**) Flow cytometric analysis depicting the proportion of NK1.1^+^ cells, CD4^+^ T cells, and CD8^+^ T cells in CD45^+^ cells in tumors harvested from mice after the indicated treatments (*n =* 5/group). Tumors are indicated by white arrows in PET images. All numerical data are presented as mean ± SD. *P < 0.05, **P < 0.01, ****P < 0.0001 by 2-way ANOVA (**B**), 2-tailed paired Student’s *t* test (**D** and **F**), and 2-tailed unpaired Student’s *t* test (**G**).

To confirm that pseudoprogression occurs owing to immune cell infiltration into the tumor and to further demonstrate the specific targeting of ^68^Ga-grazytracer to the activated immune microenvironment, we used FTY720 — an inhibitor of T cell egress from lymphoid tissues (37, 38) — to prevent lymphocyte infiltration into the tumor site (Figure 5A). FTY720 treatment partially reversed the antitumor effect of anti–PD-1 plus anti–CTLA-4 (Figure 5B) without influencing body weight (Supplemental Figure 11), demonstrating the role of CTL tumor infiltration in the antitumor effects of ICIs.

^18^F-FDG and ^68^Ga-grazytracer PET imaging experiments were then performed in the anti–PD-1 plus anti–CTLA-4 groups with or without the addition of FTY720. ^18^F-FDG showed similar imaging patterns from days 0 to 6 in the groups with or without FTY720 treatment (Figure 5, C and D). In contrast, while increased tumor uptake of ^68^Ga-grazytracer was observed from days 0 to 6 following anti–PD-1 plus anti–CTLA-4 treatment, no significant differences were observed following treatment with anti–PD-1 plus anti–CTLA-4 and FTY720 (Figure 5, E and F). Immunofluorescence staining of MC38 tumors on day 6 confirmed that FTY720 treatment reduced granzyme B secretion in tumors (Supplemental Figure 12). These results indicated that ^68^Ga-grazytracer effectively identified tumor pseudoprogression by monitoring the infiltrated and activated immune cells in the tumor microenvironment via granzyme B targeting.

Ex vivo flow cytometric analysis also showed that levels of NK, CD4^+^ T, and CD8^+^ T cells in the anti–PD-1 plus anti–CTLA-4 plus FTY720 group were significantly lower than those in the anti–PD-1 plus anti–CTLA-4 group without FTY720 (Figure 5G), further confirming the role of FTY720 in the inhibition of immune cell tumor infiltration.

### ^68^Ga-grazytracer PET enables early prediction of ACT therapy efficacy.

**Figure 6:**
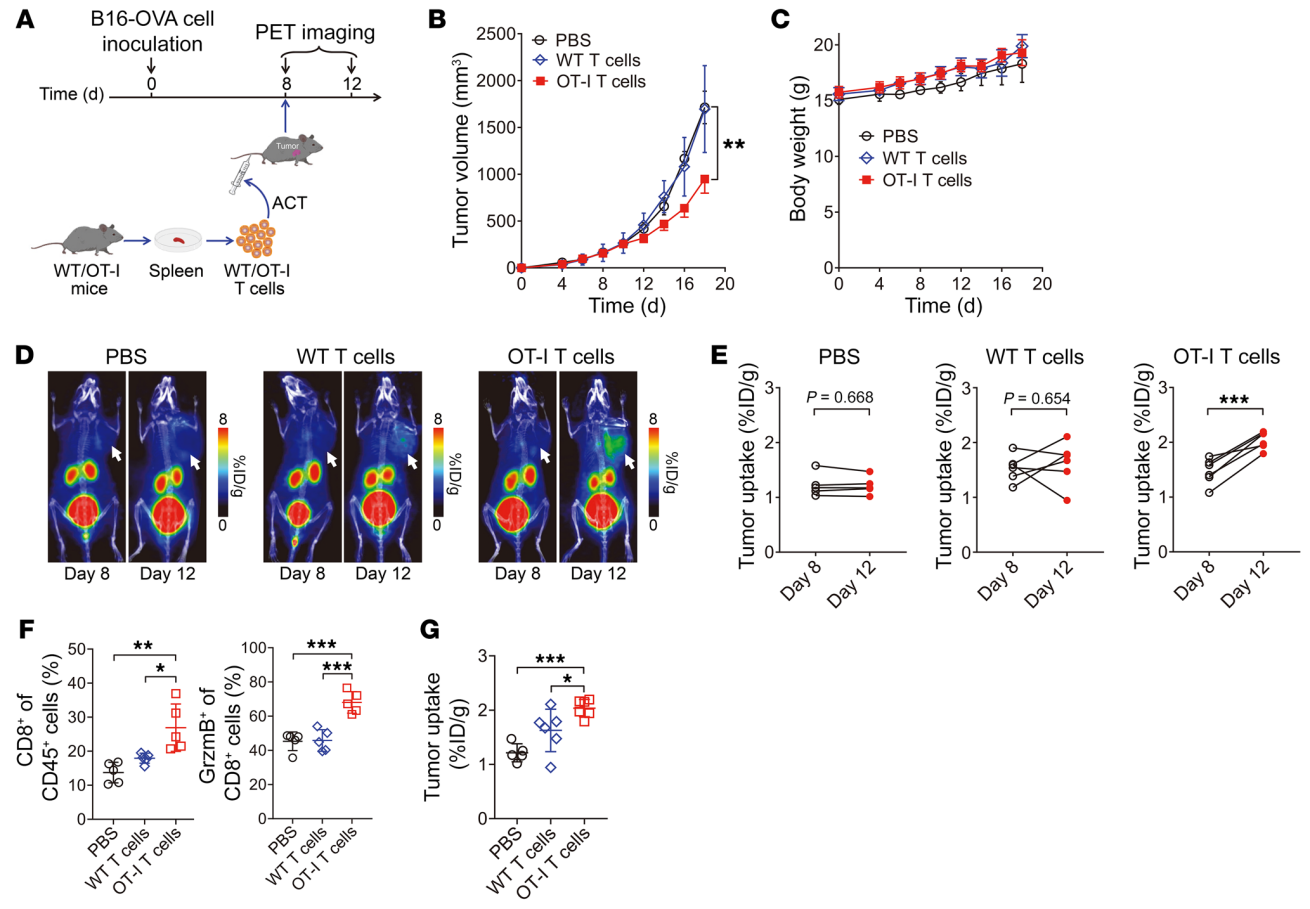
PET imaging of ^68^Ga-grazytracer in mice treated with ACT. (**A**) Schematic of PET imaging and ACT in B16-OVA tumor–bearing mice. (**B** and **C**) Tumor growth curves (**B**) and body weight (**C**) of B16-OVA tumor–bearing mice after the indicated treatments: control (PBS); and adoptive transfer of WT T cells and OT-I T cells (*n =* 5–7/group). (**D** and **E**) Representative PET images (**D**) and quantified tumor uptake (**E**) of ^68^Ga-grazytracer on days 8 and 12 in B16-OVA tumor–bearing mice treated with PBS or T cells from WT or OT-I mice (*n =* 5–6/group). Tumors are indicated by white arrows. (**F**) Flow cytometric analysis depicting the proportion of CD8^+^ T cells in CD45^+^ cells and granzyme B^+^CD8^+^ T cells in tumors harvested from mice after the indicated treatments on day 12 (*n =* 5/group). (**G**) Quantified tumor uptake of ^68^Ga-grazytracer on day 12 in mice after the indicated treatments (*n =* 5–6/group). All numerical data are presented as mean ± SD. *P < 0.05, **P < 0.01, ***P < 0.001 by 2-way ANOVA (**B**), 2-tailed paired Student’s *t* test (**E**), and 1-way ANOVA with post hoc Tukey’s test (**F** and **G**).

We further explored whether ^68^Ga-grazytracer PET could be expanded to monitor the efficacy of other granzyme B–related immunotherapies. We performed ACT studies using a B16-ovalbumin (B16-OVA) tumor–bearing mouse model. T cells from OT-I transgenic and WT mice were used for the ACT (Figure 6A). ^68^Ga-grazytracer PET was performed before (on day 8) and 4 days after treatment with ACT (on day 12) to evaluate granzyme B secretion. Compared with the control (PBS treatment), adoptively transferred OT-I T cells significantly inhibited B16-OVA tumor growth, owing to the fact that these cells specifically recognize OVA (*P* < 0.01); while transfer of WT T cells exerted minimal effects on the inhibition of tumor growth (Figure 6B). Furthermore, the body weight of mice gradually increased in all groups (Figure 6C), suggesting that ACT in mice elicited limited toxicity.

The tumor uptake of ^68^Ga-grazytracer in the control group was unchanged on day 12 compared with day 8 (Figure 6, D and E). Meanwhile, in the group that underwent adoptive transfer of WT T cells, uptake of ^68^Ga-grazytracer in some tumors increased on day 12; however, no statistical difference was observed compared with baseline values (day 8; Figure 6E). These results suggested that ACT did not produce an effective and sustained immune response without OVA-specific recognition. In contrast, significantly higher tumor uptake of ^68^Ga-grazytracer was observed in the OT-I T cell–based ACT group on day 12 compared with day 8 (*P* < 0.001; Figure 6E). Ex vivo flow cytometric analysis of B16-OVA tumors on day 12 revealed that the levels of CD8^+^ T cell infiltration and granzyme B secretion in the tumors treated with OT-I T cells were significantly higher than those in the tumors treated with PBS and WT T cells (Figure 6F). This result was consistent with the quantified tumor uptake values of ^68^Ga-grazytracer on day 12 (Figure 6G). These results indicate that OVA-specific T cells secreted the highest levels of granzyme B to kill tumor cells.

### Clinical translation of ^68^Ga-grazytracer PET in study participants.

Given the role of ^68^Ga-grazytracer PET in monitoring tumor responses to immunotherapy in animal models, we next investigated whether this radiotracer could be applied for PET in humans. Acute toxicity results in mice indicated that ^68^Ga-grazytracer was safe (Supplemental Figure 13, A–E, and Supplemental Figure 14, A–E), and the imaging dose of ^68^Ga-grazytracer did not affect tumor growth in mouse models (Supplemental Figure 15, A–C), which suggested the safety of ^68^Ga-grazytracer for further translational studies.

To assess clinical translation of the ^68^Ga-grazytracer, we enrolled 5 volunteers (4 men and 1 woman; median age, 66 years, range, 50–70 years), 3 of whom had stage IV and 2 stage III cancer, according to clinical tumor, node, metastasis (TNM) staging (Supplemental Table 1). All 5 patients underwent baseline ^18^F-FDG PET/CT before treatment and paired ^18^F-FDG PET/CT and ^68^Ga-grazytracer PET/CT imaging within 1 week of completing their indicated treatments (Supplemental Table 1). We set a lesion-to-blood pool SUV_max_ ratio greater than 1 as the standard for positive imaging results; the application of this criterion identified 2 patients with positive ^68^Ga-grazytracer PET/CT results. One of these patients (patient 1) was rated as having a PMR (by European Organization for Research and Treatment of Cancer [EORTC]), stable metabolic disease (SMD; by PERCIST), and stable disease (SD; by RECIST 1.1); the other (patient 2) was rated as PMR (by EORTC and PERCIST) and partial response (PR; by RECIST 1.1). The remaining 3 patients had negative ^68^Ga-grazytracer PET/CT results; one of them (patient 4) was rated as SMD (by EORTC and PERCIST) and SD (by RECIST 1.1); the other 2 (patients 3 and 5) were rated as PMD (by EORTC and PERCIST) and progressive disease (PD; by RECIST 1.1) (Supplemental Table 1). Thus, these findings suggest that patients with positive ^68^Ga-grazytracer PET/CT results exhibited better responses to therapy, while patients with negative results showed poorer responses.

**Figure 7:**
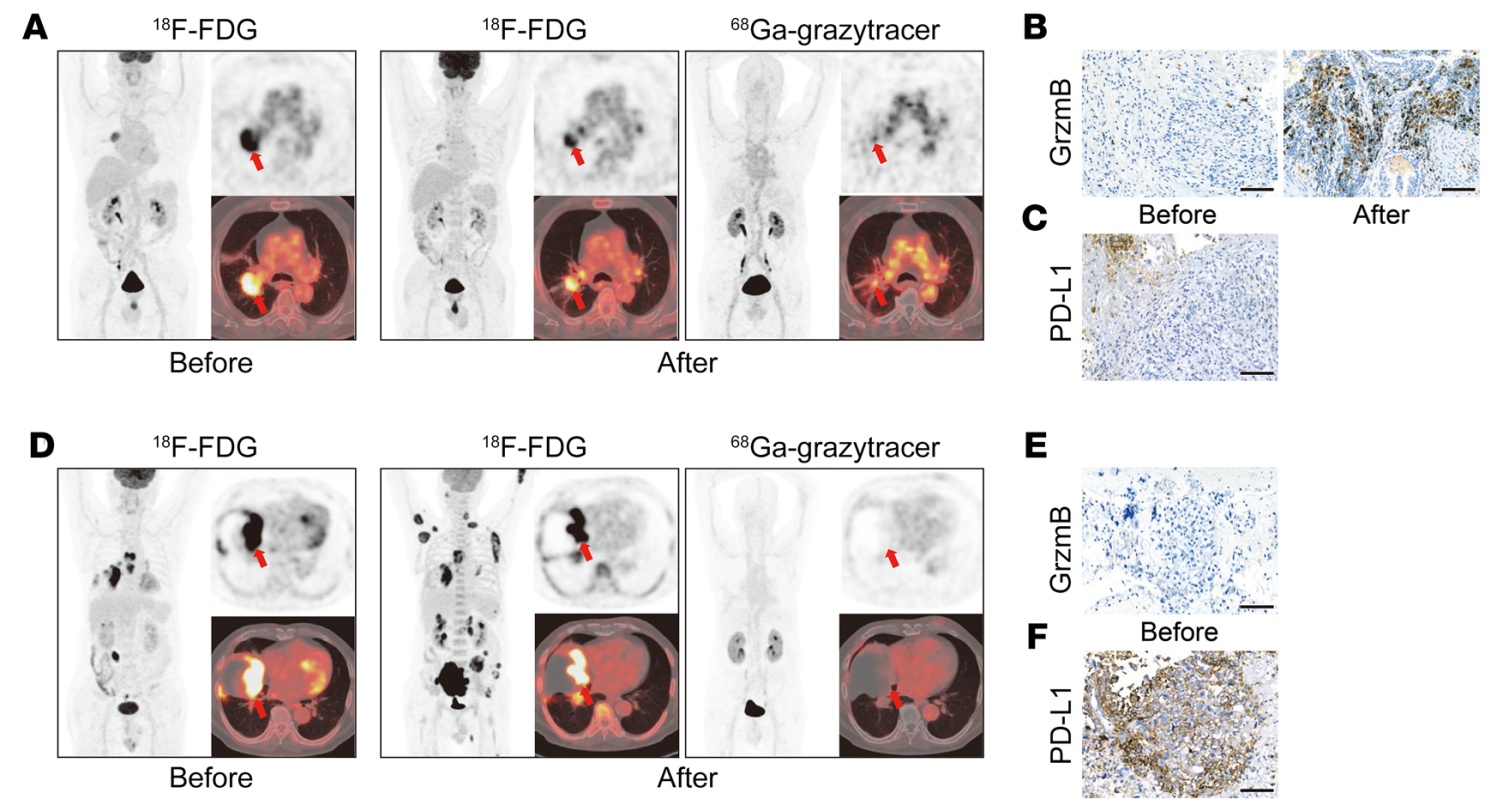
PET/CT imaging of ^68^Ga-grazytracer and ^18^F-FDG in patients with lung cancer. (**A**) A 66-year-old male (patient 1) with lung adenocarcinoma, clinical stage cT2bN2M0 (IIIa). ^18^F-FDG PET/CT before 3 cycles of chemotherapy and anti–PD-1 therapy (pemetrexed disodium + cisplatin + toripalimab) showed that the SUV_max_ was 8.5 and the SUL_peak_ was 5.3. ^18^F-FDG PET/CT after treatment showed that the SUV_max_ was 6.5 and the SUL_peak_ was 3.8; this patient was rated as PMR (with EORTC criteria) and SMD (with PERCIST criteria). ^68^Ga-grazytracer PET/CT after treatment revealed a SUV_max_ of 4.1 and tumor-to-blood pool SUV_max_ ratio (T/B ratio) of 1.2, and the patient was assessed as having positive results. (**B**) IHC staining of granzyme B in the tumor of patient 1 before and after treatment. (**C**) IHC staining of PD-L1 in the tumor of patient 1 before treatment. Scale bars: 100 μm. (**D**) A 70-year-old male (patient 3) with sarcomatoid carcinoma of the lung, clinical stage cT4N3M1c (IVb). ^18^F-FDG PET/CT before 1 cycle of pembrolizumab revealed a SUV_max_ of 39.3 and SUL_peak_ of 25.0. ^18^F-FDG PET/CT after treatment revealed a SUV_max_ of 26.4 and SUL_peak_ of 16.6; this patient was rated as PMD (with EORTC and PERCIST criteria). ^68^Ga-grazytracer PET/CT after treatment showed that the SUV_max_ was 2.0 and T/B ratio was 0.8, and the patient was assessed as having negative results. (**E** and **F**) IHC staining of granzyme B (**E**) and PD-L1 (**F**) in the tumor of patient 3 before treatment. Scale bars: 100 μm. Primary tumors are indicated by the red arrows in PET images.

In the representative case (patient 1; Supplemental Table 1) with positive ^68^Ga-grazytracer PET/CT results after 3 cycles of chemotherapy plus anti–PD-1 immunotherapy, ^18^F-FDG revealed decreased tumor uptake in the follow-up PET/CT examination (Figure 7A). IHC results confirmed markedly increased granzyme B expression in the tumor after therapy (Figure 7B), whereas IHC of the tumor before therapy showed low PD-L1 expression levels (Figure 7C).

In the representative case (patient 3; Supplemental Table 1) with negative ^68^Ga-grazytracer PET/CT results after 1 cycle of pembrolizumab treatment, ^68^Ga-grazytracer imaging showed low tumor uptake in the whole body, indicating that there was no granzyme B secretion in any of the lesions. These results suggested that patient 3 was not responsive enough to the treatment regimen to fully activate T cells, thus leading to whole-body tumor metastasis, as confirmed by ^18^F-FDG PET/CT (Figure 7D). Granzyme B IHC analysis revealed negative granzyme B expression before immunotherapy (Figure 7E). For ethical reasons, the tumor tissue of this patient could not be obtained after immunotherapy. Although IHC showed high tumor PD-L1 expression before treatment (Figure 7F), this patient did not respond to pembrolizumab after 1 treatment cycle (Supplemental Table 1). These findings implied that PD-L1, as a biomarker, may not be sufficient for predicting the efficacy of anti–PD-1 immunotherapy (39).

## Discussion

Noninvasive imaging methods capable of precisely guiding clinical decision-making would markedly improve immunotherapy efficacy, as only a small subset of patients benefit from such treatment. In this study, we designed, synthesized, and optimized a granzyme B–targeting PET radiotracer, ^68^Ga-grazytracer, that can specifically detect and quantify granzyme B secretion by effector T cells upon ICI and ACT therapy. ^68^Ga-grazytracer PET allowed discrimination between true tumor progression and pseudoprogression in mouse models, thus highlighting its potential advantages over ^18^F-FDG PET. Moreover, a translational ^68^Ga-grazytracer study in a small cohort revealed the promise for use of this radiotracer in clinical settings to monitor early tumor responses to immunotherapy.

Certain challenges limit the application of currently identified biomarkers — such as PD-L1, tumor mutational burden (TMB) (40), and gene expression profile (GEP) score (41) — as predictors of tumor responses to immunotherapies. For instance, biopsy-based biomarkers such as PD-L1 lack accuracy primarily due to limited tissue sampling and tumor heterogeneity, while analysis of biomarkers using blood samples lacks lesion specificity and does not provide whole-body information. Noninvasive PET imaging–based methods may offer an alternative approach; however, selecting optimal targeting markers is crucial considering the complexity of tumor responses to immunotherapy.

Given the role of granzyme B in the exertion of CTL function during immunotherapy, a granzyme B-targeting radiotracer, ^68^Ga-NOTA-GZP (25, 26), and its analog, ^18^F-AlF-mNOTA-GZP (42, 43), have recently been developed for noninvasive monitoring of tumor responses to ICIs in preclinical studies. Compared with ^68^Ga-NOTA-GZP, ^68^Ga-grazytracer exhibited significantly enhanced tumor uptake and tumor imaging contrast in tumor-bearing mice. This could be due to the improved in vivo metabolic stability of ^68^Ga-grazytracer, as the rigid tricyclic peptidomimetic scaffold and 1,2,3-triazole pharmacophore of ^68^Ga-grazytracer are more resistant to hydrolytic and enzymatic cleavage (44). The favorable in vivo pharmacokinetics and metabolic stability of ^68^Ga-grazytracer should assure its safe use in the clinical setting, and our preliminary study in 5 cancer patients supported the role of ^68^Ga-grazytracer PET in monitoring immunotherapy. However, owing to the limited number of patients recruited, rigorous clinical conclusions cannot be drawn so far. Large cohort studies are currently underway to validate these preliminary clinical results.

^68^Ga-grazytracer PET could be implemented in several ways. First, as the strategy of combining therapies such as chemotherapy and radiotherapy with immunotherapy is increasingly used to enhance efficacy against primary and metastatic lesions (45, 46), ^68^Ga-grazytracer PET may provide a robust platform for in vivo longitudinal monitoring of granzyme B levels during immunotherapy. This noninvasive platform would allow for the rational design of combination therapies and facilitate elucidation of the underlying mechanisms, accelerating the development of new immunotherapeutic drugs. Second, compared with current clinical imaging methods (such as CT, MRI, and ^18^F-FDG PET/CT used for noninvasive monitoring of ICI therapy), which take 4–8 weeks to confirm the presence of PD (8), ^68^Ga-grazytracer PET can identify granzyme B release upon immunotherapy, facilitating early prediction of tumor responses to therapy and early identification of pseudoprogression. Moreover, ^68^Ga-grazytracer PET together with ^18^F-FDG PET/CT could provide a complementary profile to annotate the exertion of T cell effector function and glucose metabolism in the tumor. Third, ^68^Ga-grazytracer PET could also be expanded to monitor tumor responses to other immunotherapies related to granzyme B secretion, such as chimeric antigen receptor–T (CAR-T) and CAR-NK cell therapies. Furthermore, considering that the pathology of many other conditions — including transplant rejection (47, 48), immune-mediated myocarditis (49), and other irAEs induced by immunotherapies (50) — involve granzyme B secretion, ^68^Ga-grazytracer could also be expanded for PET imaging of other disorders.

Granzyme B and perforin are transported to target cells through immune synapses that are transiently formed between cytotoxic T cells and tumor cells (51). Hence, there is an optimal period of time for detecting granzyme B secreted from effector T cells that have not yet been transported into tumor cells, which can be effectively achieved by ^68^Ga-grazytracer PET. Although we can employ murine models to explore the secretion pattern of granzyme B to determine the optimal imaging time points of ^68^Ga-grazytracer, this may require further optimization in clinical settings. Therefore, baseline scanning and longitudinal PET may be necessary to capture the secretion windows of granzyme B in the clinic to provide accurate information on T cell effector function.

In summary, a PET radiotracer, ^68^Ga-grazytracer, was designed to specifically target granzyme B and monitor the efficacy of multiple immunotherapies in different tumor mouse models. ^68^Ga-grazytracer was more effective than ^18^F-FDG in discriminating tumor pseudoprogression in animal models treated with ICIs. A preliminary clinical study further confirmed the role of ^68^Ga-grazytracer PET in monitoring immunotherapy, demonstrating theoretical feasibility for future clinical studies with larger cohorts. The straightforward synthesis procedures for ^68^Ga-grazytracer make it possible for widespread clinical application by kit formulation, and ^68^Ga-grazytracer PET may provide a general immune-monitoring approach capable of guiding precise tumor immunotherapy through tumor response prediction and patient stratification. Furthermore, as granzyme B secretion is a characteristic of various immune-related disorders, ^68^Ga-grazytracer PET may prove effective in contexts beyond tumor imaging.

## Methods

### Synthesis of granzyme B–targeting precursors.

Detailed synthesis procedures and characterization of granzyme B–targeting precursors are described in the supplemental material.

### ^68^Ga radiolabeling.

^68^GaCl_3_ was eluted from a ^68^Ge/^68^Ga generator (Isotope Technologies Garching GmbH) with 0.1N HCl. For ^68^Ga radiolabeling, 30 nmol granzyme B precursors was dissolved in 300 μL 0.1 M sodium acetate buffer (pH 5.5) and mixed with 555 MBq ^68^GaCl_3_. The mixtures were reacted for 10 minutes at 99°C and then purified using Sep-Pak C18 cartridges (Waters). After passage through 0.22 μm filters (Millipore), the respective final radiotracers were generated. The radiochemical purity of the radiotracers was determined using analytical radio-HPLC. The in vitro stability of ^68^Ga-labeled radiotracers was determined by incubation in PBS or FBS. At 0, 30, 60, and 120 minutes, the radiochemical purity of the radiotracers was determined using analytical radio-HPLC.

### In vitro granzyme B binding assay.

Granzyme B–coated plates were prepared by coating 100 ng murine granzyme B protein or human granzyme B protein (R&D Systems) onto 96-well Stripwell ELISA plates (Costar) overnight at 4°C. After washing with PBS and blocking with 1% BSA (in PBS), 74 kBq ^68^Ga-grazytracer was added to the plates, with or without the excess doses of precursor 3. After incubation for 1 hour at room temperature, the plates were washed with PBS. Wells on the plates were then collected, and the associated radioactivity was measured using a γ-counter (Packard).

### Cell culture and animal models.

The MC38 murine colon carcinoma, LLC, 4T1 murine breast cancer, and B16-OVA murine melanoma cell lines were purchased from ATCC. MC38, LLC, and B16-OVA cells were cultured in DMEM, and 4T1 cells were cultured in RPMI-1640 medium (Invitrogen). All cells were grown in medium supplemented with 10% FBS at 37°C in a humidified atmosphere containing 5% CO_2_.

Female C57BL/6 mice and BALB/c mice (5–6 weeks of age) were purchased from the Department of Laboratory Animal Science of Peking University. Female OT-I transgenic mice (5–6 weeks of age) were purchased from Shanghai Model Organisms Center Inc. To establish the MC38, LLC, and B16-OVA tumor–bearing mouse models, we inoculated 2 × 10^6^ tumor cells subcutaneously into the right upper flanks of C57BL/6 mice. To establish the 4T1 tumor–bearing mouse model, we inoculated 2 × 10^6^ 4T1 cells subcutaneously into the right upper flanks of BALB/c mice. Tumor growth was measured using a caliper every other day, and tumor volume was calculated using the formula volume = length × width^2^/2.

### Small-animal PET imaging.

Small-animal PET scanning and image analysis were performed using a Super Nova PET/CT scanner (PINGSENG). The MC38, LLC, 4T1, or B16-OVA tumor–bearing mice were administered 5.55 MBq ^68^Ga-labeled radiotracers or ^18^F-FDG under isoflurane anesthesia. For ^18^F-FDG imaging, MC38 and 4T1 tumor–bearing mice were fasted for 6 hours before receiving the ^18^F-FDG injection. At the indicated times (0.5, 1, and 2 hours) after injection, 10-minute static PET scans were acquired. PET images were analyzed, and the region of interest–derived (ROI-derived) %ID/g values were calculated as described previously (52).

### Autoradiography.

^64^CuCl_2_ was produced in a Sumitomo HM-20 biomedical cyclotron via the ^64^Ni(p,n)^64^Cu reaction. Precursor 3 was radiolabeled with ^64^CuCl_2_ using the same protocol as that for ^68^Ga-grazytracer described above. The resulting radiotracer, ^64^Cu-grazytracer (18.5 MBq), was intravenously injected into MC38 tumor–bearing C57BL/6 mice. At 0.5 hours after injection, mice were euthanized and tumors were harvested. Each tumor sample was immediately frozen in OCT medium and cut into 15 μm thick continuous slices for autoradiography of ^64^Cu-grazytracer and immunofluorescence staining of granzyme B. Autoradiography was performed using the Cyclone Plus storage phosphor system (PerkinElmer). The neighboring slices were stained for granzyme B as described below.

### Western blotting.

After small-animal PET imaging, the MC38 tumor–bearing C57BL/6 mice were euthanized, and tumor tissues were harvested. After homogenization, tumor tissue proteins were extracted, and protein concentrations were determined using a bicinchoninic acid protein assay kit (Thermo Fisher Scientific). The proteins were separated using SDS-PAGE and transferred onto a PVDF membrane (Invitrogen), blocked in 5% skim milk for 2 hours, and incubated overnight at 4°C with an anti–murine granzyme B primary antibody (1:1000; catalog 17215S, Cell Signaling Technology). Bands were visualized using the Molecular Imager PharosFX Plus System (Bio-Rad Laboratories) after incubation with HRP-conjugated secondary antibody (1:10000; A0208, Beyotime Biotechnology) for 1 hour at room temperature.

### ELISA of granzyme B.

After the MC38 tumor–bearing C57BL/6 mice were euthanized, blood and tumors were harvested. The proteins were extracted by incubating with cold tissue extraction buffer (0.5 mL) containing protease inhibitors (Cwbio). Levels of granzyme B in the tumor lysates and serum were determined using a murine granzyme B ELISA kit (Dogesce Corp.) following the manufacturer’s instructions. With the assumption that the total blood volume of a C57BL/6 mouse (body weight ~20 g) was 2 mL, the fraction of circulating granzyme B in the bloodstream was estimated as previously described (53).

### In vivo anti–PD-1 or anti–PD-1 plus anti–CTLA-4 therapy.

MC38 tumor–bearing C57BL/6 mice were treated with an intraperitoneal injection of PBS (vehicle control) or 200 μg anti–PD-1 antibody (clone RMP1-14, BioXcell) on days 0, 3, and 6. LLC-bearing C57BL/6 mice were treated with PBS (vehicle control) or 200 μg anti–PD-1 plus 100 μg anti–CTLA-4 antibodies (clone 9D9, BioXcell) on days 0, 3, and 6. Mice were subjected to PET imaging at 0.5 hours following intravenous injection of ^68^Ga-grazytracer. Flow cytometric analysis of CD8, IFN-γ, and granzyme B was performed on day 9.

### In vivo depletion studies.

For the in vivo depletion of CD4^+^ T, CD8^+^ T, or NK cells, anti-CD8 antibody (400 μg; clone YTS 169.4, BioXcell), anti-CD4 antibody (400 μg; clone GK1.5, BioXcell), or anti-NK1.1 antibody (400 μg; clone PK136, BioXcell) was administered twice weekly via intraperitoneal injection into MC38 tumor–bearing C57BL/6 mice 1 day before the first treatment with anti–PD-1 antibody (clone RMP1-14, BioXcell; 5 doses of 200 μg every 3 days).

### PET imaging in pseudoprogression and true progression mouse models.

For the pseudoprogression and true progression tumor models, MC38 and 4T1 tumor–bearing mice were treated with an intraperitoneal injection of 200 μg anti–PD-1 (clone RMP1-14, BioXcell) plus 200 μg anti–CTLA-4 (clone 9D9, BioXcell) antibodies on days 0, 3, and 6. On day 0 (baseline) and day 6, mice were subjected to small-animal PET imaging of ^68^Ga-grazytracer or ^18^F-FDG at 0.5 or 1 hour after injection.

### FTY720 treatment.

To evaluate the role of infiltrating T cells in the tumor uptake of ^68^Ga-grazytracer, MC38 tumor–bearing C57BL/6 mice were intraperitoneally injected with 25 μg of FTY-720 (Cayman Chemical) on day –1 and continuous administration of FTY-720 (10 μg daily) was performed from day 0 to day 12. Mice were also treated with three doses of anti–PD-1 (clone RMP1-14; BioXcell; 200 μg × 3) plus anti–CTLA-4 (clone 9D9; BioXcell; 200 μg × 3) antibodies on days 0, 3, and 6. On day 0 (baseline) and day 6, mice were subjected to small-animal PET imaging of ^68^Ga-grazytracer or ^18^F-FDG at 0.5 or 1 h postinjection. On day 6, five mice from each group were euthanized, and the tumor tissues were harvested. Each tumor was digested to obtain single-cell suspensions and subsequently sorted for NK1.1, CD4, and CD8 by flow cytometry as described below.

### Adoptive T cell transfer studies.

Spleens from OT-I mice were disrupted and passed through a 70 μm cell strainer. After lysis of red blood cells using ammonium-chloride-potassium (ACK) lysis buffer (Macgene), splenocytes were resuspended at a cell density of 1 × 10^6^/mL in RPMI-1640 medium supplemented with mouse IL-2 (10 ng/mL; PeproTech), IL-7 (1 ng/mL, PeproTech), and OVA_257–264_ peptide (1 μM; Yuanye Bio-Technology). After 3 days of culture, live cells were enriched by density gradient centrifugation (GE Healthcare), and the cells were resuspended in complete RPMI-1640 medium containing mouse IL-2 (10 ng/mL) and IL-7 (10 ng/mL) to reach a cell density of 0.5 × 10^6^/mL. Cells were then cultured for another 2 days. T cell acquisition from WT mice was performed using the same protocol, except that additional antibodies — anti-CD3 (clone 145-2C11; 1 μg/mL, BioLegend) and anti-CD28 (clone 37.51; 2 μg/mL, BioLegend) — were included in the first 3-day culture.

When the tumor size reached 200 mm^3^, B16-OVA tumor–bearing C57BL/6 mice were treated with adoptive T cells (5 × 10^6^ per mouse) from WT or OT-I mice. Tumor growth was monitored, and mice were subjected to small-animal PET imaging of ^68^Ga-grazytracer at 0.5 hours after injection on days 8 and 12. On day 12, five mice from each group were euthanized, and their tumor tissues were harvested for flow cytometric analysis of CD8 and granzyme B.

### Flow cytometric analysis.

MC38 or B16-OVA tumor–bearing C57BL/6 mice were euthanized, and tumor tissues were harvested and digested with 10 U/mL collagenase I, 400 U/mL collagenase IV, and 30 U/mL DNase (Yuanye Bio-Technology) to generate single-cell suspensions. The following fluorescently labeled antibodies were then used for staining of different markers: anti-CD45 (clone 30-F11, BioLegend), anti-CD4 (clone RM4-5, eBioscience), anti-CD8 (clone 53-6.7, BioLegend), anti-NK1.1 (clone PK136, BioLegend), anti–granzyme B (clone QA16A02, BioLegend), and anti–IFN-γ (clone XMG1.2, BioLegend). For granzyme B and IFN-γ staining, cells were incubated in culture medium containing a cell activation cocktail (catalog 423303, BioLegend) at 37°C for 5 hours and then washed with permeabilization wash buffer (catalog 421002, BioLegend) before being stained with fluorescently labeled antibodies. Data collection and analysis were performed on a flow cytometer (Becton Dickinson).

### Immunofluorescence staining.

The tumor sections were fixed with ice-cold acetone for 10 minutes and washed with PBS. Next, the slices were blocked with 1% BSA for 1 hour and incubated with rabbit anti–mouse granzyme B antibody (catalog 17215S, Cell Signaling Technology) or rabbit anti–mouse NK1.1 antibody (clone EPR22990-31, Abcam) overnight at 4°C. The slices were then visualized by incubation with FITC-conjugated secondary antibody (catalog E031220-01, Earthox) and DAPI (Biotium) under a confocal microscope (Leica).

### PET/CT imaging of study participants.

A retrospective review was performed from ^18^F-FDG PET/CT scans obtained in routine clinical practice in patients before and after ICI therapy, and an illustrative case was selected for this study. A total of 5 patients (4 with lung cancer and 1 with melanoma of rectal mucosa) who received immunotherapy were enrolled in this study. Evaluation of the therapeutic response was based on the PERCIST (54), EORTC (55), and RECIST 1.1 (56) standards. The patients underwent PET/CT using a Siemens Biograph mCT Flow 64 scanner. Patients fasted for 4–6 hours before receiving an intravenous injection of ^18^F-FDG (5.55 MBq/kg of body weight); no specific preparation was requested of participants before the intravenous injection of ^68^Ga-grazytracer (3.7 MBq/kg of body weight; chemical dose ~0.22 nmol/kg). Low-dose CT scanning was performed with the same parameters (120 kV; 146 mÅ; slice: 3 mm; matrix: 200 × 200; iterations: 2; subsets: 11; filter: 5 mm Gaussian), and PET acquisitions were performed 40 ± 10 minutes after ^68^Ga-grazytracer injection or 1 hour after ^18^F-FDG injection. Imaging was performed by continuously moving the patient bed at a speed of 1.5 mm/s from the top of the skull to the upper femur. After scanning, the images were reconstructed using ordered subset expectation maximization. CT reconstruction used a standard method, with a 512 × 512 matrix and a layer thickness of 3–5 mm. CT data were used to correct the PET images for attenuation; and PET, CT, and fusion images of the cross-section, sagittal plane, and coronal plane were obtained. Images were processed using a Siemens workstation (syngo.via client 4.1).

For PET/CT image processing and analysis, 3 nuclear medicine physicians analyzed the images independently, and the diagnosis was made only when 2 or more physicians agreed. The ROIs were outlined; and SUV_max_, SUV was normalized to body surface area (SUV_BSA_), and SUL_peak_ were recorded.

### IHC.

Patient tumor samples from biopsy or surgery were obtained from Peking University Cancer Hospital. After antigen retrieval, tumor sections were stained for PD-L1 using a pharmDx kit (clone 22C3, Agilent Technologies) according to the manufacturer’s instructions. For granzyme B staining, the tumor sections were incubated with anti–human granzyme B antibody (1:200; clone EP230, ZSGB-BIO) overnight at 4°C. Tumor sections were then incubated with an HRP-conjugated secondary antibody for 2 hours and visualized following incubation with diaminobenzidine substrate.

### Statistics.

Statistical analyses were performed using GraphPad Prism 9 software. Quantitative data are presented as mean ± SD. Data obtained from 2 groups were analyzed using a 2-tailed unpaired Student’s *t* test. The comparisons of tumor uptake of radiotracers before and after indicated treatments were performed using a 2-tailed paired Student’s *t* test. One-way ANOVA with a post hoc Tukey’s test was used to compare multiple groups. Tumor growth curves over time were compared using 2-way ANOVA. The correlation between 2 variables was determined using standard Pearson’s correlation analysis. *P* values less than 0.05 were considered statistically significant.

### Study approval.

All animal experiments were carried out according to protocols approved by the Peking University Animal Care and Use Committee. Clinical imaging and all other studies involving participants were approved by the Institutional Review Board of Peking University Cancer Hospital (approval no. 2021KT86). All participants provided written informed consent. This trial was registered at ClinicalTrials.gov (NCT05000372).

## Author contributions

YW, HYZ, and ZL designed the experiments. HYZ and YW performed most of the preclinical experiments and analyzed data. TZ, YZ, KL, and HZ assisted with animal studies and data analysis. XY supervised the precursor synthesis. HX synthesized and characterized the precursors. NL and ZY supervised the clinical studies. XS, XZ, and NL performed the clinical imaging studies and analyzed data. LZ performed the IHC of clinical samples. HYZ, YW, and ZL prepared the figures. HYZ, YW, XY, NL, and ZL wrote the manuscript with contributions from the other authors. ZL conceived the project and supervised the research. All authors reviewed and approved the final version of the manuscript.

## Supplementary Material

Supplemental data

## Version 1 07/05/2022

In-Press Preview

## Version 2 08/15/2022

Electronic publication
