# Supplementary material for: Noninvasive interrogation of CD8+ T cell effector function for monitoring early tumor responses to immunotherapy
Source: J Clin Invest. 2022 Aug 15;132(16):e161065. doi: 10.1172/JCI161065 (PMC9374377; doi:10.1172/JCI161065)
Supplement: Supplemental data [file jci-132-161065-s024.pdf]

Supplementary Information for

Noninvasive interrogation of CD8$^+$ T cell effector function for monitoring tumor early responses to immunotherapy

Haoyi Zhou[1†], Yanpu Wang[1†], Hongchuang Xu[2], Xiuling Shen[3], Ting Zhang[1], Xin Zhou[3], Yuwen Zeng[1], Kui Li[1], Li Zhang[4], Hua Zhu[3], Xing Yang[2*], Nan Li[3*], Zhi Yang[3], Zhaofei Liu[1*]

[1]Medical Isotopes Research Center and Department of Radiation Medicine, School of Basic Medical Sciences, Peking University Health Science Center, Beijing 100191, China

[2]Department of Nuclear Medicine, Peking University First Hospital, Beijing 100034, China

[3]Key Laboratory of Carcinogenesis and Translational Research (Ministry of Education/Beijing), Department of Nuclear Medicine, Peking University Cancer Hospital & Institute, Beijing 100142, China

[4]Department of Pathology, Peking University Cancer Hospital & Institute, Beijing 100142, China

[†]These authors contributed equally to this work.

*Address correspondence to: Zhaofei Liu, 38 Xueyuan Road, Beijing 100191, China. Phone: 86-10-82805825; E-mail: liuzf@bjmu.edu.cn; or Xing Yang, 8 Xishiku Street, Beijing 100034, China. Phone: 86-10-83572928; Email: yangxing2017@bjmu.edu.cn; or Nan Li, 52 Fucheng Road, Beijing 100142, China. Phone: 86-10-88196364; Email: rainbow6283@sina.com.

This file includes:

Supplementary Materials and Methods

Supplementary References

Figures S1 to S15

Table S1

Supplementary Materials and Methods

Synthesis of granzyme B-targeting precursors

All reagents and solvents were commercially available and used without further purification unless otherwise noted.

1. Synthesis of 2,2',2''-(10-(2-((8-(((S)-3-(((2S,3S)-1-(((3S,6S)-6-(((2H-1,2,3-triazol-4-yl)methyl)carbamoyl)-4-oxo-1,2,3,4,6,7-hexahydroazepino[3,2,1-hi]indol-3-yl)amino)-3-methyl-1-oxopentan-2-yl)amino)-1-carboxy-3-oxopropyl)amino)-8-oxooctyl)amino)-2-oxoethyl)-1,4,7,10-tetraazacyclododecane-1,4,7-triyl)triacetic acid (**Precursor 1**).

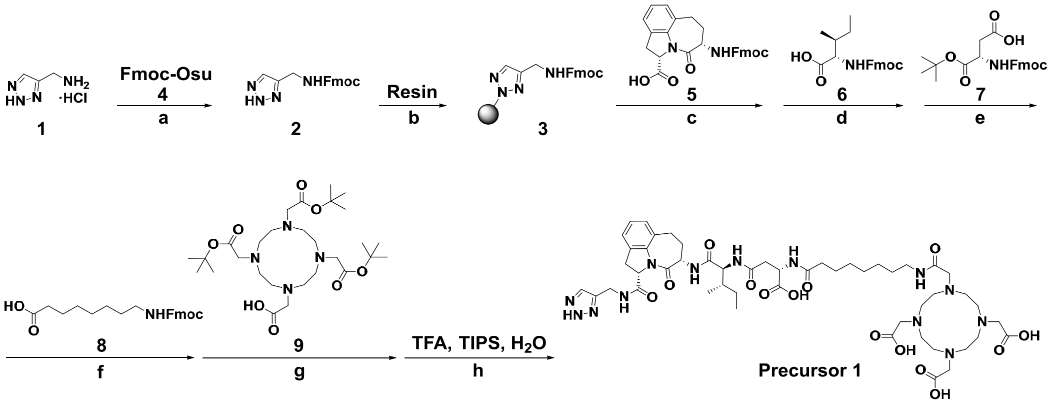

Scheme 1. Synthesis route for **Precursor 1**. Reagents and solvents used are as follows: a) compound **4**, Et_3N, DCM; b) 2-Chlorotrityl chloride resin, DIPEA, DCM/DMF; c) compound **5**, HBTU, HOBT, DIPEA, DMF; d) compound **6**, HBTU, HOBT, DIPEA, DMF; e) compound **7**, HBTU, HOBT, DIPEA, DMF; f) compound **8**, HBTU, HOBT, DIPEA, DMF; g) compound **9**, HBTU, HOBT, DIPEA, DMF; h) TFA, TIPS, H_2O.

*Synthesis of (9H-fluoren-9-yl)methyl ((2H-1,2,3-triazol-4-yl)methyl)carbamate (Compound **2**)*

Compound **1** (134 mg, 1.0 mmol), Fmoc-Osu (337 mg, 1.0 mmol), and triethylamine (Et_3N, 121 mg, 1.2 mmol) were mixed and stirred in dichloromethane

(DCM, 10 mL) at room temperature for 30 min. The solvent was removed by evaporation under reduced pressure. 272 mg of compound **2** was obtained by flash column chromatography (DCM/MeOH, 10/1) as a white solid with a yield of 85%. ESI-MS (m/z): 321.05 (calc. 321.13, $[C_{18}H_{16}N_4O_2]H^+$).

*Loading compound **2** to resin (3)*

2-Chlorotrityl chloride resin (1.0 g) was swollen in DCM (10 mL) at room temperature for 1 h. The resin was then mixed with compound **2** (160 mg, 0.5 mmol) and diisopropylethylamine (DIPEA, 175 μL, 1.0 mmol) in dimethylformamide (DMF)/DCM (1/1, 4 mL) and stirred at room temperature for 2 h. After the solvent was removed, the resin was washed with DCM/MeOH/DIPEA (10/10/1, 7 mL × 5 min × 3). Resin **3** was filtered as a yellow solid loaded with 0.4 mmol of compound **2**.

*Synthesis of **Precursor 1**.*

Resin **3** (100 mg, 0.04 mmol) was swollen in DCM (2 mL × 5 min × 3) and washed with DMF (2 mL × 5 min × 3). Next, solid-phase synthesis was carried out according to the following repeated procedures:

1) The resin was stirred in 20% piperidine DMF solution (2 mL × 10 min × 2) to remove the Fmoc protecting group and washed with DMF (2 mL × 2 min × 5). After deprotection, the resin was mixed and stirred with compound **5** (112 mg, 0.24 mmol), which was preactivated for 15 min with HBTU (91 mg, 0.24 mmol), HOBT (32 mg, 0.24 mmol), and DIPEA (50 μL, 0.29 mmol) in DMF (3 mL). The reaction mixture was stirred at room temperature for 2 h, and the resin was then washed with DMF (2 mL × 2 min × 5).

2)–5) Following a similar procedure as described in step 1), compound **6** (85 mg,

0.24 mmol), compound **7** (99 mg, 0.24 mmol), compound **8** (92 mg, 0.24 mmol), and compound **9** (137 mg, 0.24 mmol) were sequentially conjugated to the resin.

6) The final product was cleaved from the resin in TFA/triisopropylsilane (TIPS)/H_2O (95/2.5/2.5, 5 mL) for 2 h. After high-performance liquid chromatography (HPLC) purification, 7.1 mg **Precursor 1** was obtained as a white solid with a 16% yield and 98% purity. HRMS (m/z): 1082.5610 (calc. 1082.5629, $[C_{50}H_{75}N_{13}O_{14}]H^+$).

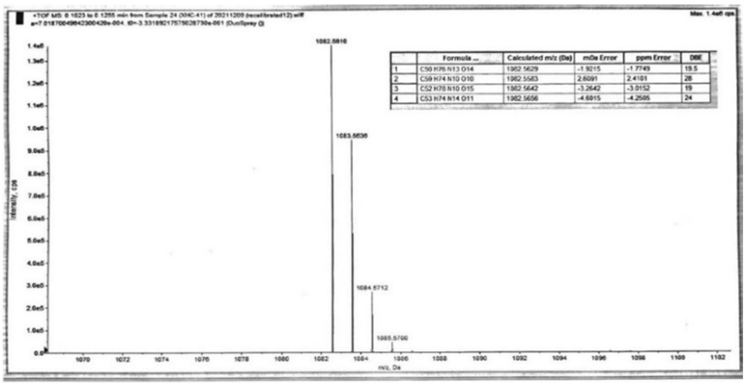

The HRMS of **precursor 1**

2. Synthesis of 2,2',2''-(10-(2-((3-(2-(((2S,3S)-1-(((3S,6S)-6-(((2H-1,2,3-triazol-4-yl)methyl)carbamoyl)-4-oxo-1,2,3,4,6,7-hexahydroazepino[3,2,1-hi]indol-3-yl)amino)-3-methyl-1-oxopentan-2-yl)amino)-2-oxoethyl)benzyl)amino)-2-oxoethyl)-1,4,7,10-tetraazacyclododecane-1,4,7-triyl)triacetic acid (**Precursor 2**).

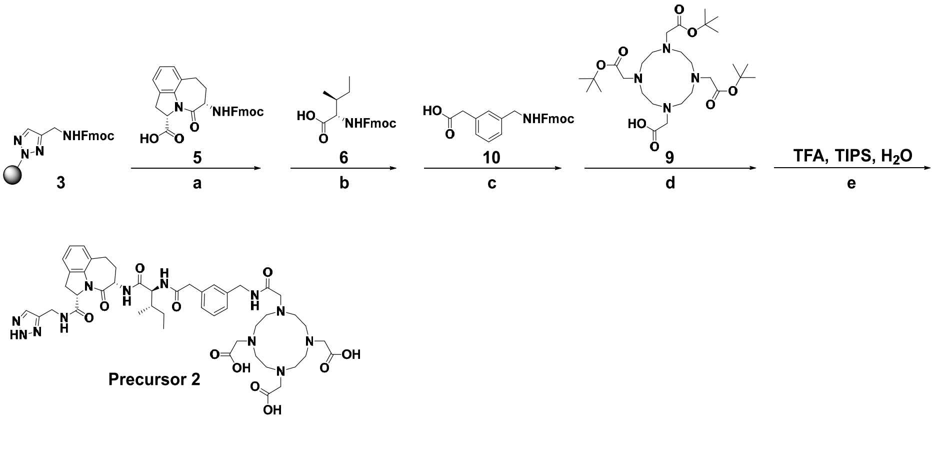

Scheme 2. Synthesis route for **precursor 2**. Reagents and solvents used are as follows:

a) compound **5**, HBTU, HOBT, DIPEA, DMF; b) compound **6**, HBTU, HOBT, DIPEA, DMF; c) compound **10**, HBTU, HOBT, DIPEA, DMF; d) compound **9**, HBTU, HOBT, DIPEA, DMF; e) TFA, TIPS, H_2O.

Similar to the synthesis of **Precursor 1**, compound **5** (112 mg, 0.24 mmol), compound **6** (85 mg, 0.24 mmol), compound **10** (93 mg, 0.24 mmol), and compound **9** (137 mg, 0.24 mmol) were conjugated sequentially to resin **3** (100 mg, 0.04 mmol). The product was cleaved from the resin in TFA/TIPS/H_2O (95/2.5/2.5, 5 mL) for 2 h. After HPLC purification, 8.4 mg **Precursor 2** was obtained as a white solid with a 22% yield and 97% purity. HRMS (m/z): 973.4887 (calc. 973.4890, $[C_{47}H_{64}N_{12}O_{11}]H^+$).

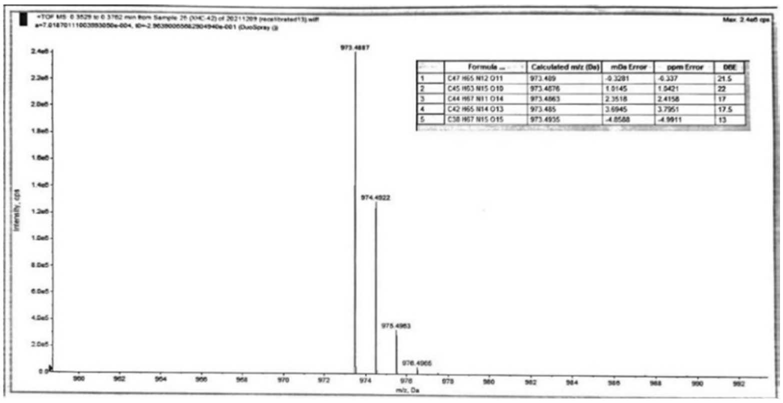

The HRMS of **precursor 2**

3. Synthesis of 2,2',2''-(10-(2-(((S)-3-(((S)-3-((3-(2-(((2S,3S)-1-(((3S,6S)-6-(((2H-1,2,3-triazol-4-yl)methyl)carbamoyl)-4-oxo-1,2,3,4,6,7-hexahydroazepino[3,2,1-hi]indol-3-yl)amino)-3-methyl-1-oxopentan-2-yl)amino)-2-oxoethyl)benzyl)amino)-1-carboxy-3-oxopropyl)amino)-1-carboxy-3-oxopropyl)amino)-2-oxoethyl)-1,4,7,10-tetraazacyclododecane-1,4,7-triyl)triacetic acid (**Precursor 3**).

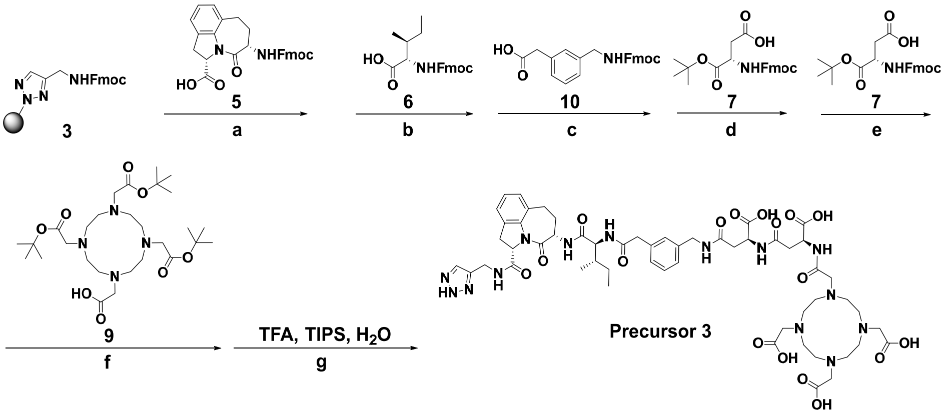

Scheme 3. Synthesis route for **Precursor 3**. Reagents and solvents used are as follows: a) compound **5**, HBTU, HOBT, DIPEA, DMF; b) compound **6**, HBTU, HOBT, DIPEA, DMF; c) compound **10**, HBTU, HOBT, DIPEA, DMF; d) compound **7**, HBTU, HOBT, DIPEA, DMF; e) compound **7**, HBTU, HOBT, DIPEA, DMF; f) compound **9**, HBTU, HOBT, DIPEA, DMF; g) TFA, TIPS, H_2O.

Similar to the synthesis of **Precursor 1**, compound **5** (112 mg, 0.24 mmol), compound **6** (85 mg, 0.24 mmol), compound **10** (93 mg, 0.24 mmol), compound **7** (99 mg, 0.24 mmol), compound **7** (99 mg, 0.24 mmol), and compound **9** (137 mg, 0.24 mmol) were conjugated sequentially to resin **3** (100 mg, 0.04 mmol). The product was cleaved from the resin in TFA/TIPS/H_2O (95/2.5/2.5, 5 mL) for 2 h. After HPLC purification, 12 mg **precursor 3** was obtained as a white solid with a 25% yield and 98% purity. HRMS (m/z): 1203.5430 (calc. 1203.5429, $[C_{55}H_{74}N_{14}O_{17}]H^+$).

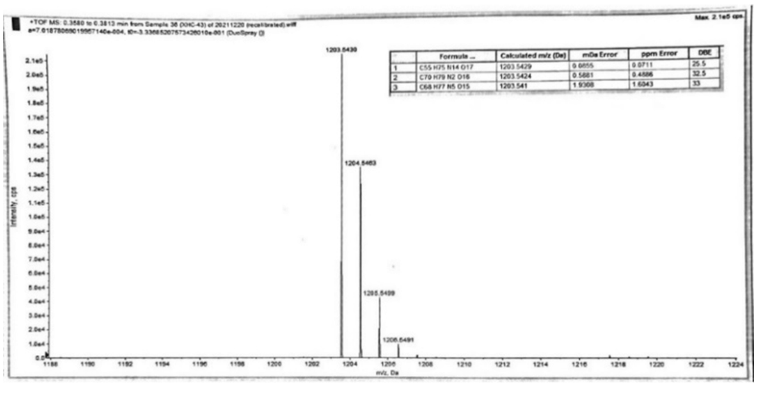

The HRMS of **precursor 3**

4. Synthesis of 2,2',2''-(10-(2-(((S)-3-((3-(2-(((2S,3S)-1-(((3S,6S)-6-(((2H-1,2,3-triazol-4-yl)methyl)carbamoyl)-4-oxo-1,2,3,4,6,7-hexahydroazepino[3,2,1-hi]indol-3-yl)amino)-3-methyl-1-oxopentan-2-yl)amino)-2-oxoethyl)benzyl)amino)-1-carboxy-3-oxopropyl)amino)-2-oxoethyl)-1,4,7,10-tetraazacyclododecane-1,4,7-triyl)triacetic acid (**Precursor 4**).

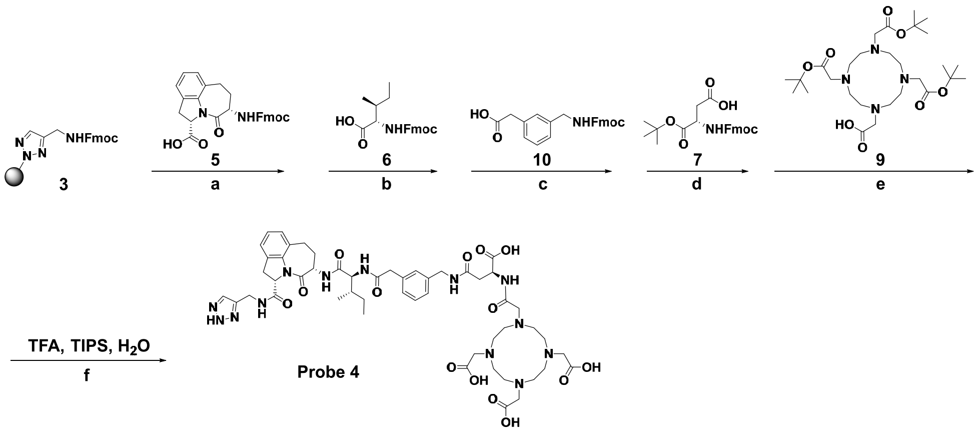

Scheme 4. Synthesis route for **Precursor 4**. Reagents and solvents used are as follows: a) compound **5**, HBTU, HOBT, DIPEA, DMF; b) compound **6**, HBTU, HOBT, DIPEA, DMF; c) compound **10**, HBTU, HOBT, DIPEA, DMF; d) compound **7**, HBTU, HOBT, DIPEA, DMF; e) compound **9**, HBTU, HOBT, DIPEA, DMF; f) TFA, TIPS, H_2O.

Similar to the synthesis of **Precursor 1**, compound **5** (112 mg, 0.24 mmol), compound **6** (85 mg, 0.24 mmol), compound **10** (93 mg, 0.24 mmol), compound **7** (99 mg, 0.24 mmol), and compound **9** (137 mg, 0.24 mmol) were conjugated sequentially to resin **3** (100 mg, 0.04 mmol). The product was cleaved from the resin in TFA/TIPS/H_2O (95/2.5/2.5, 5 mL) for 2 h. After HPLC purification, 11 mg **Precursor 4** was obtained as a white solid with a 25% yield and 98% purity. HRMS (m/z): 1088.5168 (calc. 1088.5159, $[C_{51}H_{69}N_{13}O_{14}]H^+$).

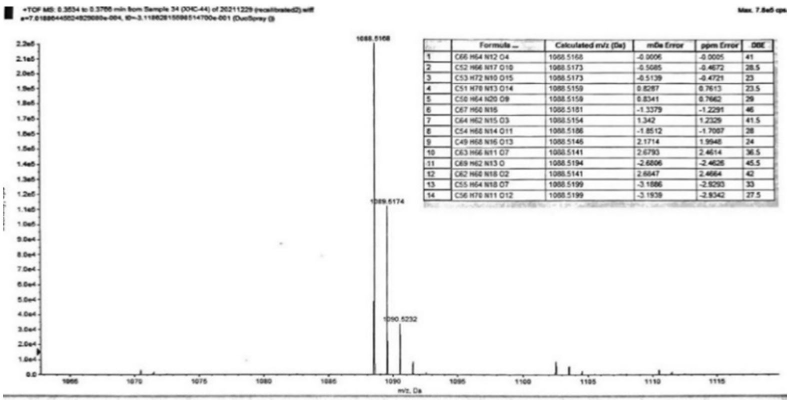

The HRMS of **precursor 4**

Biodistribution studies

MC38 tumor-bearing C57BL/6 mice were pretreated with anti-PD-1 antibody (clone RMP1-14; BioXcell, West Lebanon, NH) via intraperitoneal injection (200 μg/day on days 0, 3, and 6). The mice were then injected with 0.37 MBq of radiotracer 1, radiotracer 2, radiotracer 3 (^{68}Ga-grazytracer), or radiotracer 4 to evaluate the distribution of the radiotracer in the main organs. Mice were euthanized at 0.5, 1, and 2 h postinjection, and blood, tumor, main organs, and tissues were harvested, weighed, and measured using a γ counter (Packard, Meriden, CT). The results are presented as the percent injected dose per gram of tissue (%ID/g).

In vivo metabolic stability studies

Female C57BL/6 normal mice were intravenously injected with 5.55 MBq of ^{68}Ga-grazytracer or ^{68}Ga-NOTA-GZP. At 0.5 h postinjection, serum and urine samples were collected. After centrifugation, the supernatant was diluted with an aqueous solution of 50% acetonitrile, filtered through a 0.22-μm Millipore filter (Millipore, Billerica, MA), and analyzed using radio-HPLC.

Synthesis and characterization of ^{68}Ga-NOTA-GZP

NOTA-GZP was kindly gifted by Prof. Shaoli Song at the Fudan University Shanghai Cancer Center. ^{68}Ga-NOTA-GZP was prepared using the same protocol as described previously (1). The in vivo metabolic stability of ^{68}Ga-NOTA-GZP was compared with that of ^{68}Ga-grazytracer in female C57BL/6 normal mice.

For the small-animal PET imaging studies, ten MC38 tumor-bearing C57BL/6 mice were pretreated with 200 μg anti-PD-1 (clone RMP1-14; BioXcell) and 200 μg anti-CTLA-4 (clone 9D9; BioXcell) antibodies on days 0, 3, and 6 after the tumor size reached ~200 mm^3. Five of the ten mice were randomly selected for injection with 5.55 MBq ^{68}Ga-NOTA-GZP, while the remaining five were injected with 5.55 MBq ^{68}Ga-grazytracer. Mice were anesthetized with 2% inhaled isoflurane, and 10-min static PET scans were acquired at 0.5, 1, and 2 h postinjection using a small-animal Super Nova PET/CT scanner (PINGSENG, Shanghai, China). The tumor %ID/g values and tumor-to-muscle ratios of ^{68}Ga-NOTA-GZP and ^{68}Ga-grazytracer were calculated for comparison.

Toxicity analysis of ^{68}Ga-grazytracer

A total of 37 MBq of ^{68}Ga-grazytracer mixed without (1×), or with 100-fold (100×), 200-fold (200×), or 500-fold (500×) cold **Precursor 3** was injected intravenously into female or male BALB/c mice. Body weight was monitored every other day. Peripheral blood was collected once or twice per week and subjected to routine blood analysis as described previously (2).

To investigate whether the imaging dose of ^{68}Ga-grazytracer affects tumor growth, MC38 tumor-bearing C57BL/6 mice were treated with an intravenous injection of 5.55 MBq of ^{68}Ga-grazytracer or phosphate-buffered saline (PBS; as a vehicle control) on

days 0, 6, and 12. Tumor sizes and body weights of the mice were measured every other day.

References

1. Jiang C, et al. Engineering a smart agent for enhanced immunotherapy effect by simultaneously blocking PD-L1 and CTLA-4. *Adv Sci (Weinh)*. 2021;8(20):e2102500.

2. Zhao Y, et al. ICAM-1 orchestrates the abscopal effect of tumor radiotherapy. *Proc Natl Acad Sci U S A*. 2021;118(14):e2010333118.

Supplementary Figures and Figure Legends

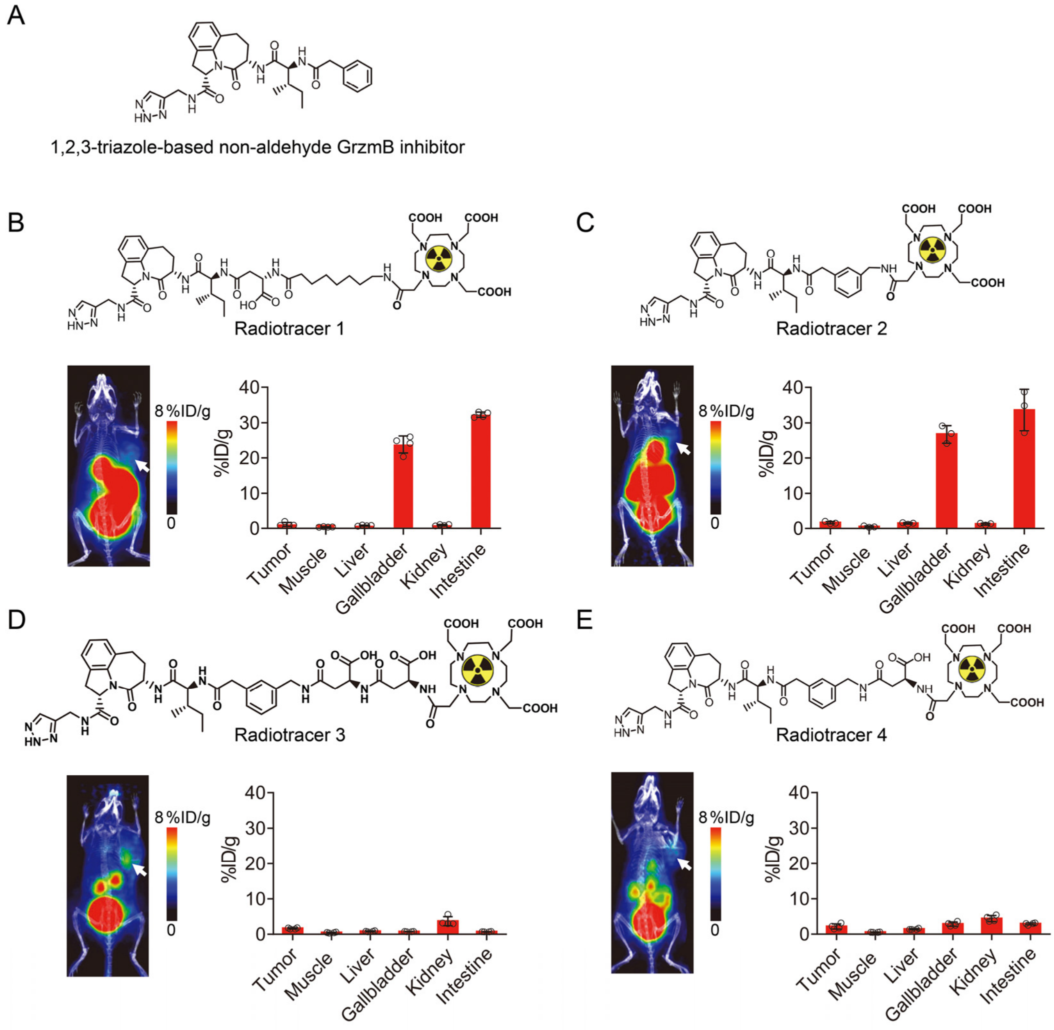

Figure S1. Chemical structure and PET imaging of four granzyme B-targeting radiotracers. (A) Chemical structure of the 1,2,3-triazole-based non-aldehyde granzyme B (GrzmB) inhibitor. (B–E) Chemical structure, representative PET images, and quantified organ uptake of radiotracer 1 (B), radiotracer 2 (C), radiotracer 3 (D), and radiotracer 4 (E) at 0.5 h postinjection in MC38 tumor-bearing mice pretreated with anti-PD-1 antibody. Tumors are indicated by white arrows in PET images. All numerical data are presented as mean ± SD, n = 3–4/group.

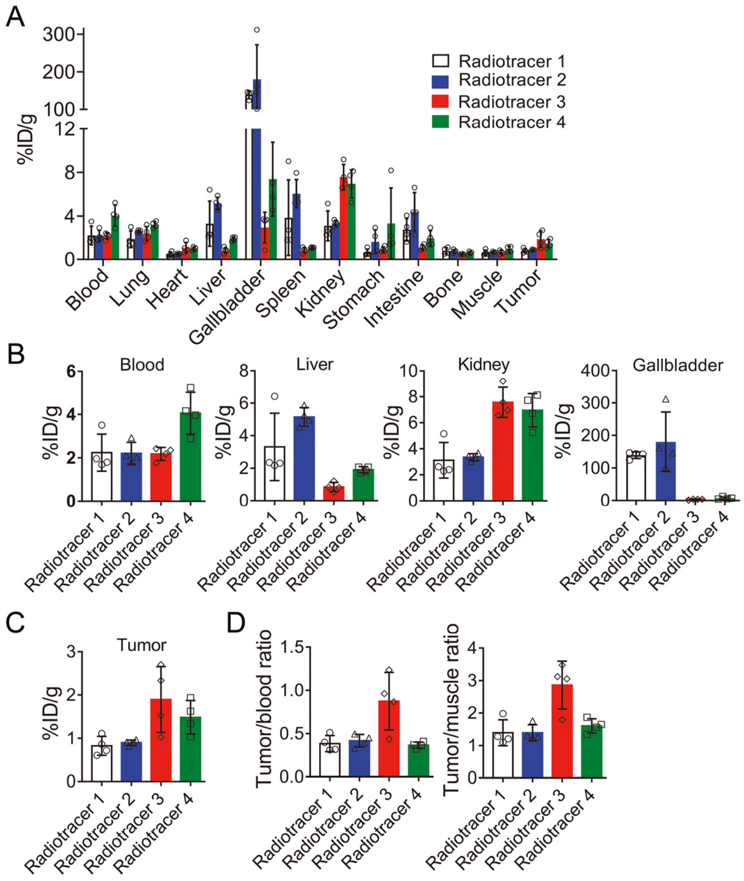

Figure S2. Biodistribution of four granzyme B-targeting radiotracers. (A) Biodistribution of radiotracer 1–4 in major organs at 0.5 h postinjection in MC38 tumor-bearing mice pretreated with anti-PD-1 antibody. (B) Uptake values of radiotracers 1–4 in the blood, liver, kidney, and gallbladder. (C) Uptake values of radiotracers 1–4 in the MC38 tumor. (D) Calculated tumor-to-blood and tumor-to-muscle ratios of radiotracers 1–4. Data are presented as mean ± SD, n = 4/group.

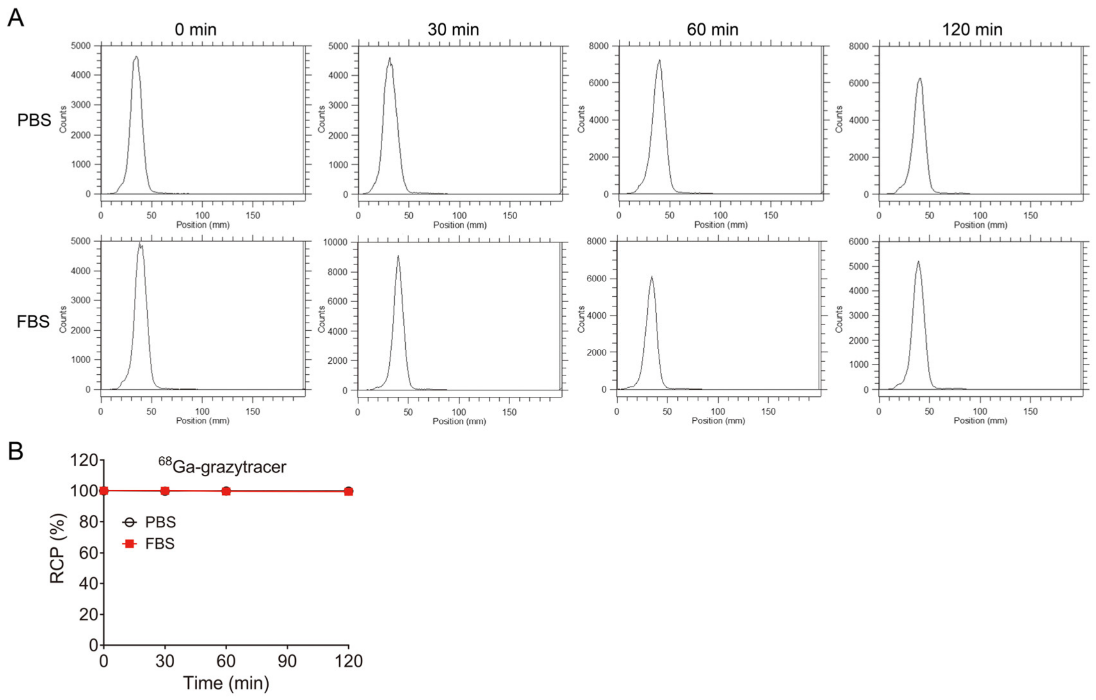

Figure S3. In vitro stability of ^{68}Ga-grazytracer. (A, B) Representative instant thin-layer chromatography (A) and radiochemical purity (RCP) values (B) of ^{68}Ga-grazytracer after incubating for 0, 30, 60, and 120 min in phosphate-buffered saline (PBS) or fetal bovine serum (FBS). Data are presented as mean ± SD, n = 3.

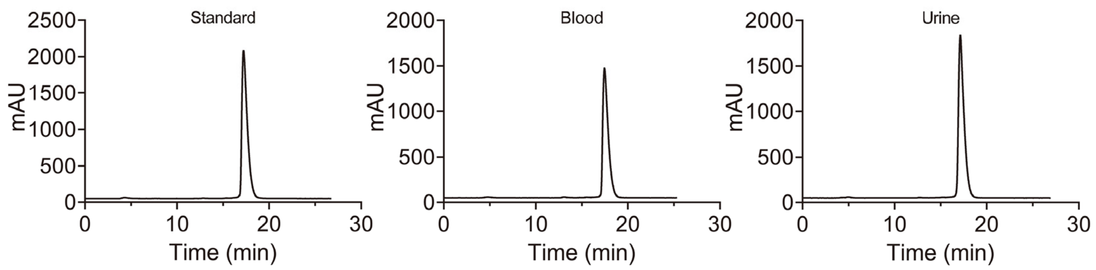

Figure S4. In vivo metabolic stability of ^{68}Ga-grazytracer. HPLC analysis of blood and urine harvested from C57BL/6 mice 0.5 h after injecting 5.55 MBq ^{68}Ga-grazytracer. Data are representative of three independent experiments.

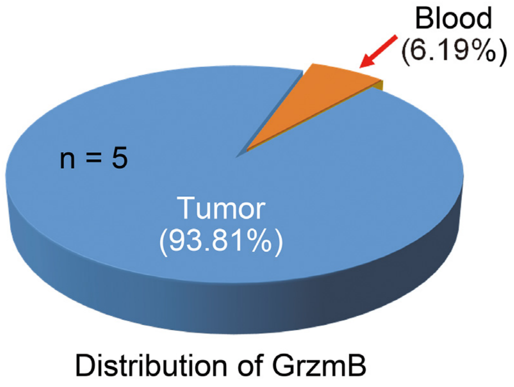

Figure S5. Comparison of granzyme B distribution in mice. The pie chart shows the distribution of granzyme B (GrzmB) in the bloodstream (6.19 ± 1.42 %) and tumors (93.81 ± 1.42 %) of anti-PD-1 antibody pretreated MC38 tumor-bearing mice (assuming that the total blood volume of a mouse is 2 mL) (n = 5).

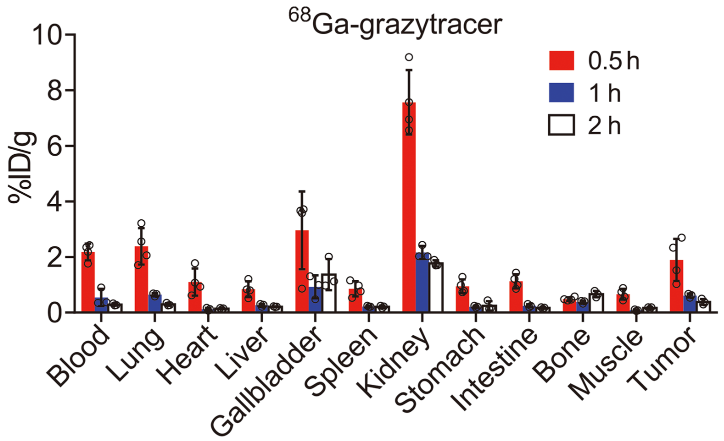

Figure S6. Biodistribution of ^{68}Ga-grazytracer. Ex vivo biodistribution of ^{68}Ga-grazytracer at 0.5, 1, and 2 h postinjection in MC38 tumor-bearing mice pretreated with anti-PD-1 antibody. Data are presented as mean ± SD, n = 3–4.

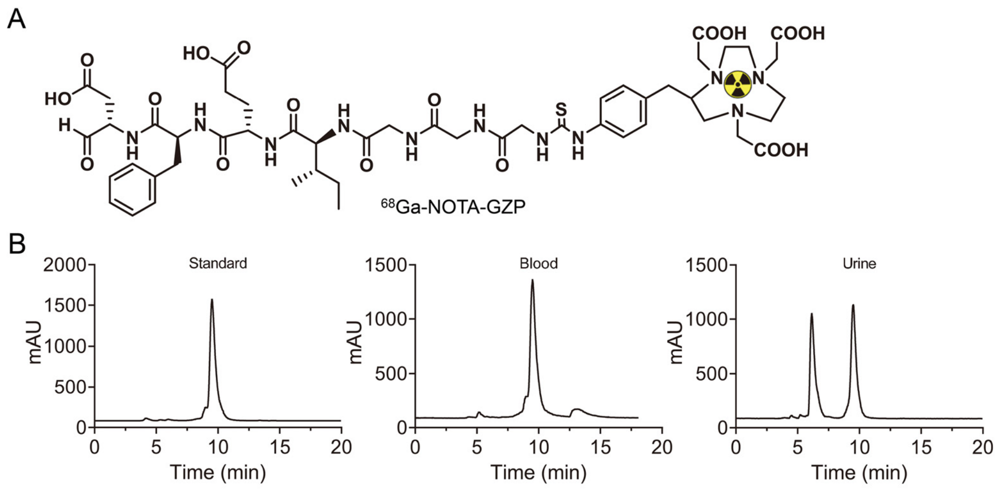

Figure S7. Chemical structure and in vivo metabolic stability of ^{68}Ga-NOTA-GZP. (A) Chemical structure of ^{68}Ga-NOTA-GZP. (B) HPLC analysis of the blood and urine harvested from C57BL/6 mice 0.5 h after injection of 5.55 MBq ^{68}Ga-NOTA-GZP. Data are representative of three independent experiments.

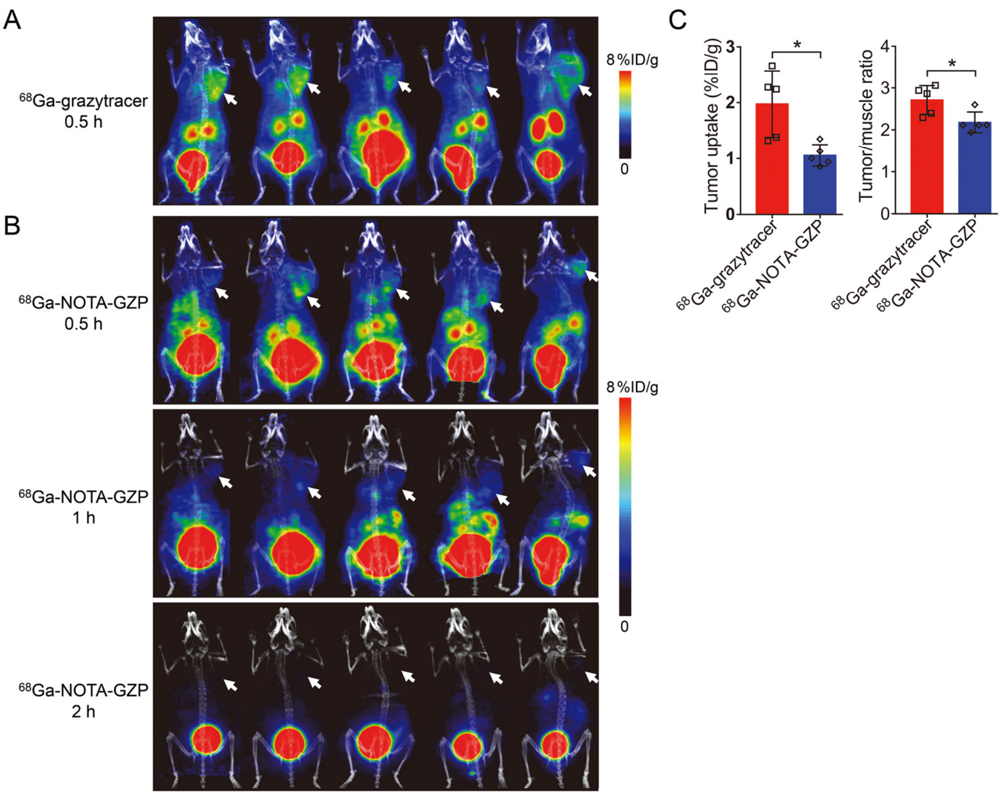

Figure S8. Comparison of ^{68}Ga-grazytracer and ^{68}Ga-NOTA-GZP PET imaging. (A) Small-animal PET images of five MC38 tumor-bearing mice pretreated with anti-PD-1 plus anti-CTLA-4 at 0.5 h postinjection of ^{68}Ga-grazytracer. (B) Small-animal PET images of five MC38 tumor-bearing mice pretreated with anti-PD-1 plus anti-CTLA-4 at 0.5, 1, and 2 h postinjection of ^{68}Ga-NOTA-GZP. Tumors are indicated by white arrows in PET images. (C) Quantified tumor uptake and tumor-to-muscle ratio of ^{68}Ga-grazytracer and ^{68}Ga-NOTA-GZP at 0.5 h postinjection. Data are presented as mean ± SD, n = 5. *, P <0.05 by unpaired Student t test (C).

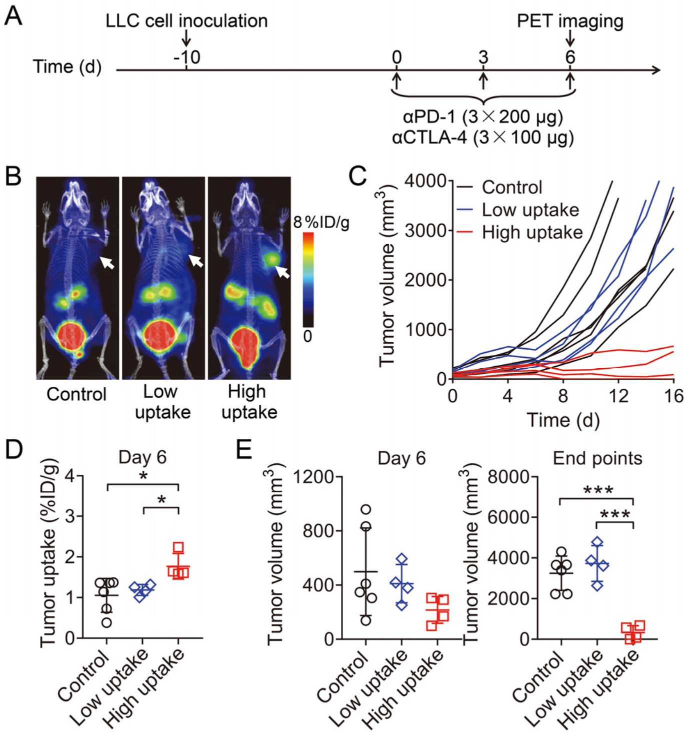

Figure S9. Small-animal PET imaging of ^{68}Ga-grazytracer to predict tumor responses to immune checkpoint blockade therapy in Lewis lung carcinoma (LLC)-bearing mice. (A) Timeline of therapy and PET imaging in LLC-bearing mice. (B) Representative PET images of ^{68}Ga-grazytracer at 0.5 h postinjection in LLC-bearing mice treated with PBS (control) or anti-PD-1 (αPD-1) plus anti-CTLA-4 (αCTLA-4) combination therapy with high and low tumor uptake (cutoff of 1.37 %ID/g). Tumors are indicated by white arrows. (C) Individual tumor volumes of LLC-bearing mice in control or treatment groups with high and low tumor uptake. (D) Quantified tumor uptake of ^{68}Ga-grazytracer at 0.5 h postinjection on day 6 in each group of LLC-bearing mice (n = 4–6/group). (E) Tumor volumes of LLC-bearing mice on day 6 and at the end points of the study (on day 16 or at the time points that mice were early euthanized owing to ethical reasons) (n = 4–6/group). All numerical data are presented as mean ± SD. *, P <0.05; ***, P <0.001 by one-way ANOVA with a post-hoc Tukey test (D, E).

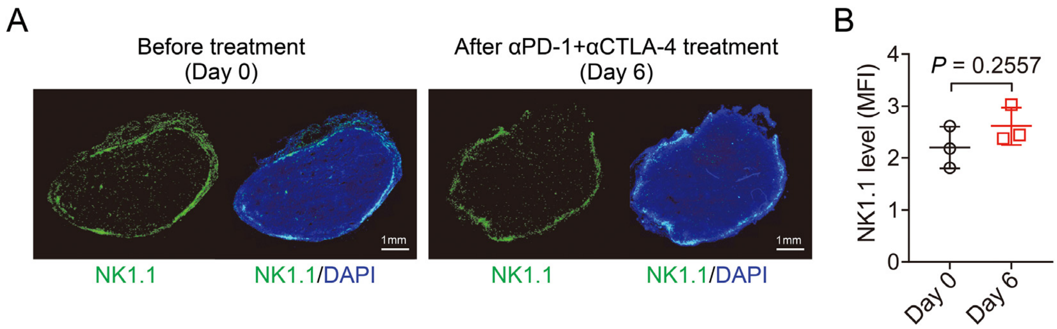

Figure S10. Infiltration level of NK cells before and after immune checkpoint blockade therapy determined using immunofluorescence staining. (A) Representative immunofluorescence staining of NK1.1 of tumor sections from MC38 tumor-bearing mice before (day 0) and after (day 6) treatment with anti-PD-1 (αPD-1) plus anti-CTLA-4 (αCTLA-4). Scale, 1 mm. (B) Quantitation of fluorescence intensity of stained NK1.1 (n = 3/group). Data are presented as mean ± SD, and were analyzed using a two-tailed unpaired Student *t* test.

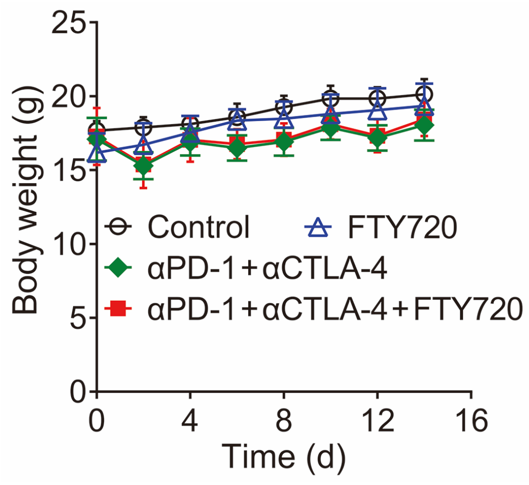

Figure S11. Body weight changes in mice. Body weight of MC38 tumor-bearing mice after the indicated treatments: control (PBS), FTY720, anti-PD-1 (αPD-1) plus anti-CLTA-4 (αCTLA-4), and αPD-1 plus αCTLA-4 plus FTY720. Data are presented as mean ± SD, n = 6–9/group. Data related to Figure 5B of the main text.

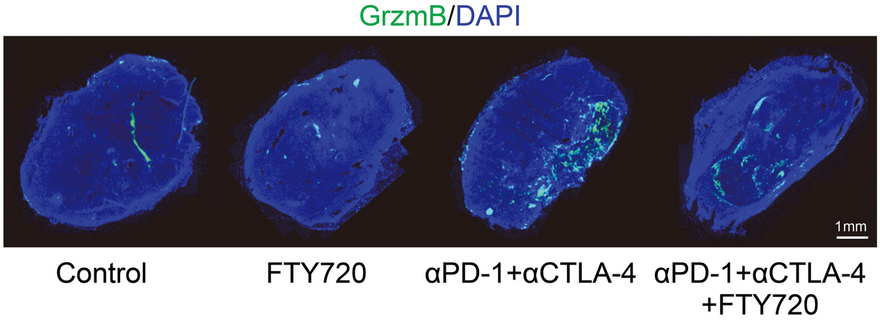

Figure S12. Representative immunofluorescence staining of granzyme B (GrzmB) of tumor sections harvested from MC38 tumor-bearing mice after indicated treatment: control (PBS), FTY720, anti-PD-1 (αPD-1) plus anti-CTLA-4 (αCTLA-4), and αPD-1 plus αCTLA-4 plus FTY720. Scale, 1 mm. Data are representative of three independent experiments.

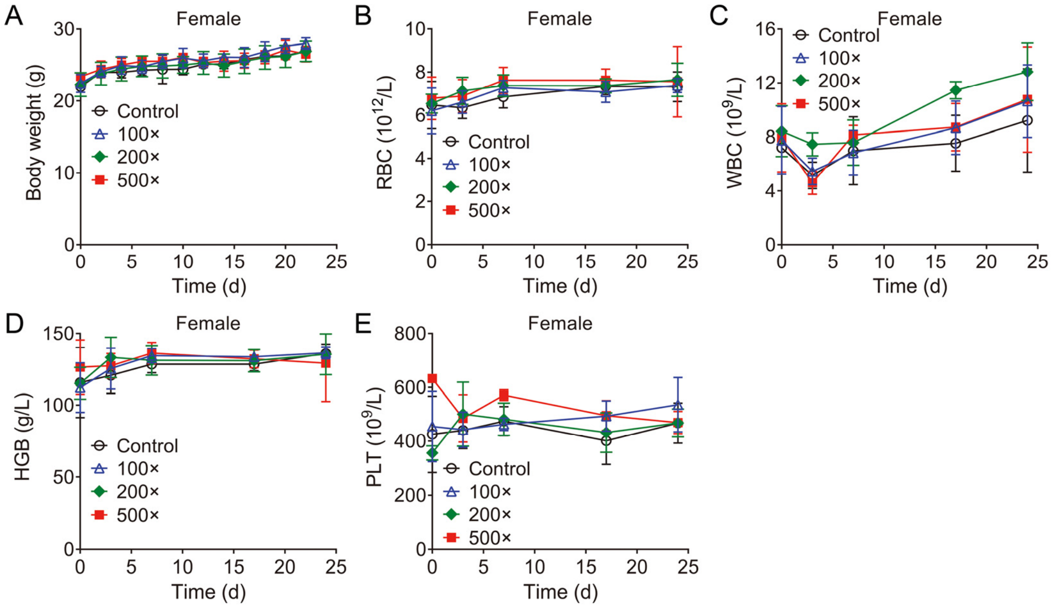

Figure S13. Acute toxicity analysis of ^{68}Ga-grazytracer in female mice. (A) Body weight, (B) red blood cells (RBC), (C) white blood cells (WBC), (D) hemoglobin (HGB), and (E) platelets (PLT) analyzed using a blood analyzer. Data are presented as mean ± SD, n = 4/group.

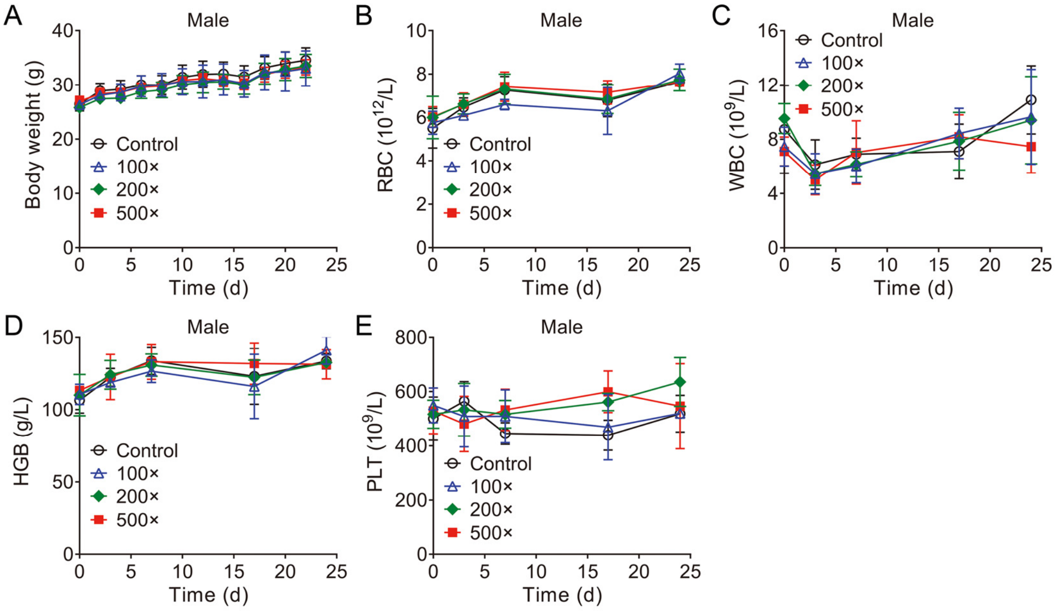

Figure S14. Acute toxicity analysis of ^{68}Ga-grazytracer in male mice. (A) Body weight, (B) red blood cells (RBC), (C) white blood cells (WBC), (D) hemoglobin (HGB), and (E) platelets (PLT) analyzed using a blood analyzer. Data are presented as mean ± SD, n = 4/group.

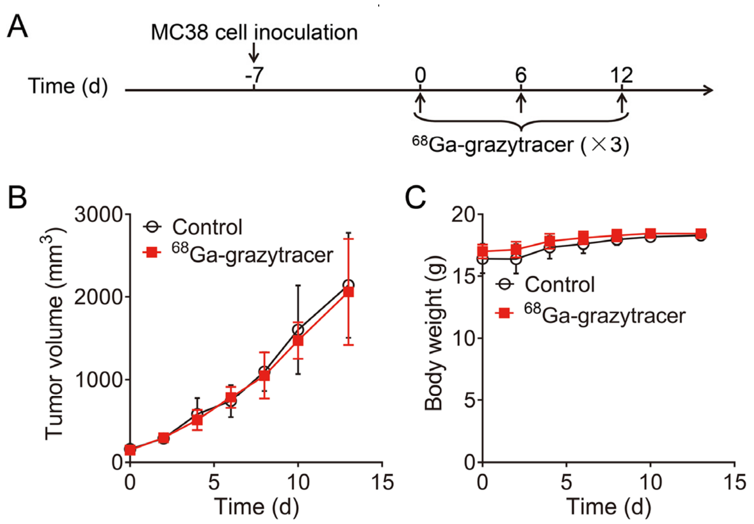

Figure S15. Effect of ^{68}Ga-grazytracer on tumor growth. (A) Timeline of ^{68}Ga-grazytracer treatment in MC38 tumor-bearing mice. (B, C) Tumor growth curves (B) and body weight (C) of MC38 tumor-bearing mice after intravenous injection of 3 doses of 5.55 MBq ^{68}Ga-grazytracer or PBS (control). Data are presented as mean ± SD, n = 8/group.

Table S1. Patient characteristics.

Patient No.	Sex	Age (Y)	Tumor Type	Stage	Therapy Regimen	^{18}F-FDG PET/CT									^{68}Ga-grazytracer PET/CT			
						Tumor size (cm)		RECIST 1.1	SUV_{BSA}		EORTC	SUL_{peak}		PERCIST	SUV_{max}	T/B	T/M	Result
						Before treatment	After treatment		Before treatment	After treatment		Before treatment	After treatment		After treatment	After treatment	After treatment	
1	Male	66	Lung adenocarcinoma	cT2bN2 M0 IIIa	Pemetrexed disodium + cisplatin + toripalimab (3 cycles)	4.4×3.0	3.5×2.0	SD	2.0	1.4	PMR	5.3	3.8	SMD	4.1	1.2	6.8	Positive
2	Male	67	Small cell lung cancer	cT2N3 M1b IVa	Etoposide + cisplatin + cindilimab + IBI110 (2 cycles)	4.6×3.1	2.8×2.3	PR	3.2	1.8	PMR	8.6	3.9	PMR	2.4	1.2	4.0	Positive
3	Male	70	Sarcomatoid carcinoma of the lung	cT4N3 M1c IVb	Pembrolizumab (1 cycle)	9.7×8.0	11.1×8.1	PD	10.2	6.9	PMD	25.0	16.6	PMD	2.0	0.8	2.9	Negative
4	Male	65	Small cell lung cancer	cT4N2 M1a IVa	Etoposide + cisplatin + durvalumab (6 cycles)	7.3×3.5	5.0×2.1	SD	3.8	3.1	SMD	9.5	7.2	SMD	1.4	0.7	2.0	Negative
5	Female	50	Melanoma of rectal mucosa	cTxN1 M0 III	JS001 + axitinib (11 cycles)	2.5×2.4	2.1×1.7	PD	3.5	4.7	PMD	7.3	8.1	PMD	2.1	0.8	3.5	Negative

Abbreviations: SUV_{BSA}, standardized uptake value normalized to body surface area; SUL_{peak}, peak standardized uptake value corrected for lean body mass; SUV_{max}, the maximum standardized uptake value; T/B, tumor-to-blood pool SUV_{max} ratio; T/M, tumor-to-muscle SUV_{max} ratio; SD, stable disease; PR, partial response; PD, progressive disease; PMR, partial metabolic response; PMD, progressive metabolic disease; SMD, stable metabolic disease.

The corresponding PET images in Figure 2G

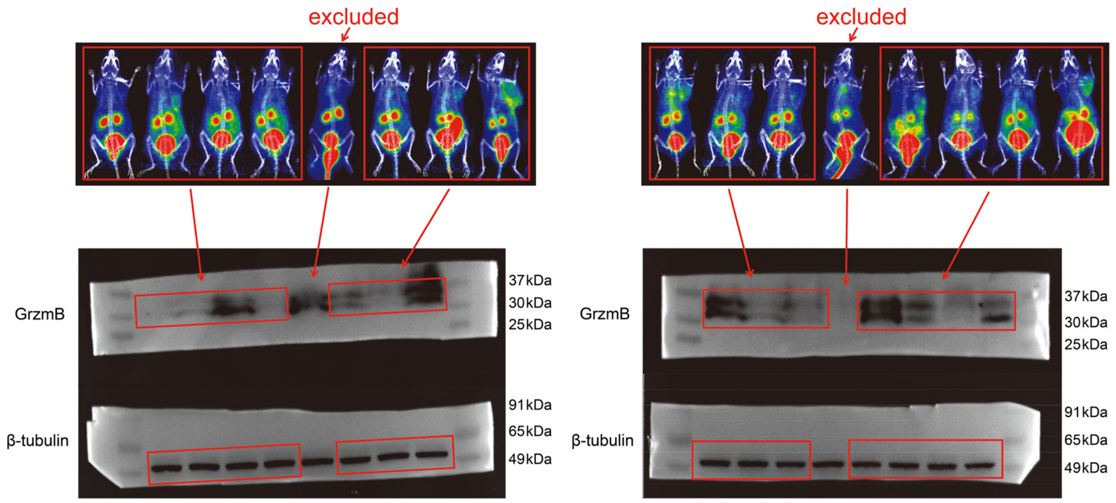

Full unedited gel for Figure 2H

Note: PET imaging and the corresponding western blotting experiments were carried out in a blinded manner. PET scanning of the MC38 tumor-bearing mice was carried out by one investigator (Y.W.). During the image reconstruction and data processing procedures (by Y.W.), all tumors were harvested from the mice immediately after PET scanning, and these samples were subjected to western blotting by another investigator (H-Y.Z.) who was blinded to the PET results. On analysis of the correlation between PET and western blotting results, two samples from mice were excluded due to failed tail vein injection and inaccurate PET quantification.
